# Children’s Drawing as a Projective Measure to Understand Their Experiences of Dental Treatment under General Anesthesia

**DOI:** 10.3390/children7070073

**Published:** 2020-07-03

**Authors:** Ziad D. Baghdadi, Saffana Jbara, Nazeem Muhajarine

**Affiliations:** 1 Dr. Gerald Niznick College of Dentistry, University of Manitoba, P131B, 780 Bannatyne Avenue, Winnipeg, MB R3E0W2, Canada; saffana.jbara@umanitoba.ca; 2 College of Medicine, University of Saskatchewan, Saskatoon, SK S7N 5E5, Canada; nazeem.muhajarine@usask.ca

**Keywords:** art, child, oral health, general anesthesia, dental care

## Abstract

Purpose: The overall aim of the study was to gain a deeper understanding of 3 to 10 year-old children’s experiences, main concerns, and how they manage attending hospital for dental treatment under general anesthesia (DTGA). Methods: Twelve children aged 3–10 who were scheduled for DTGA were interviewed. In addition to tape-recorded interviews, data were collected using video diaries, participant observations, and pre-, peri-, and postoperative drawings. The children’s drawings (*n* = 43) were analyzed using the *Child Drawing: Hospital Manual* (CD:H) and Vygotsky postulations for context readings, with the aim to explore what it means for children to undergo DTGA. Results: The analysis found that the main concern for children during the pre-operative period was that they were forced to prepare for an unknown experience, which elicited stress. This situation was handled during the peri-operative period by trying to recover control and to cooperate despite fear, stress, and anxiety. Drawings completed post-operatively showed the surgical mask, “stinky” smell of the anesthetic gas, and multiple extraction of teeth were the main troubling experiences for children. Several weeks after DTGA, children tried to regain normalcy in their lives again. Conclusion: This study contributed to a deeper understanding of how children as young as 3 years undergoing DTGA experience and express their lived experiences: emotional, psychological, physiological, or physical stress in the context of DTGA.

## 1. Introduction

Edwards (1979), author of *Drawing on the Right Side of the Brain*, wrote that, “The object of drawing is not only to show what you are trying to portray, but also to show *you*…Paradoxically, the more clearly you can perceive and draw what you see in the external world, the more clearly the viewer can see *you*, and the more you can know about *yourself*.” (pp. 23–24) [1]

Eliciting children’s opinions and thoughts about health and wellbeing is critical to making meaningful, relevant recommendations about matters affecting their lives. This recognition of children’s unique outlook is increasingly acknowledged in medical literature, particularly literature related to patient–provider communication [2]. Research has shown that healthcare providers spend more time communicating with parents than pediatric patients; in a typical medical care visit, less than 20% of the communication engaged pediatric patients, regardless of age [3]. Even more, when a provider attempts to engage a child in conversation, it is generally related to social topics, not to medical history or treatment decisions, typically completed by provider and parent [4]. Children are therefore unaccustomed to discussing their health complaints with professionals. Direct communication with a child patient builds trust and rapport, and helps to socialize children into a patient role, leading to better medical care experiences, improved outcomes, and greater satisfaction with medical interactions [5].

Drawings have been used in various ways to study children’s relationship with dentistry. For example, Taylor, Roth, and Mayberry (1976) asked 1101 children to draw a picture of a dentist at work [6]. The drawings, which were evaluated based on 60 characteristics, contained a dental chair, a dentist, a patient in the chair, and dental cabinetry or furniture. Similarly, Sheskin, Klein, and Lowental (1982) [7] used children’s drawings to evaluate anxiety in a dental setting. Aminabadi, Ghoreishizadeh, Ghoreishizadeh, and Oskouei (2011) [8] tested the hypothesis that drawing analysis can be a reliable assessment tool for evaluating a child’s distress (pain and anxiety) during conventional pediatric dental procedures. Children (ages 4–11) drew a picture of a person in a dental clinic using an A4 (8.27 × 11.69 inches) paper sheet and pencil crayons. The pictures were scored by a pediatric dentist and a psychologist based the *Child Drawing: Hospital (CD:H),* manual developed to measure anxiety in hospitalized school-age children [9,10]. Results indicated that drawing can be a projective, natural mode of communication in the field of dentistry that children rarely resist; other studies’ results [8,11,12,13] have been consistent. The overall aim of the study was to gain a deeper understanding of 3 to 10 year old children’s experiences, main concerns, and how they manage attending hospital for dental treatment under general anesthesia.

## 2. Method

The data presented here were collected as part of a larger qualitative study that examined the lived experiences of families with children who required dental treatment under general anesthesia (DTGA) [14]. Twelve families were recruited, and parents (12 mothers, 1 father) were interviewed while children (*n* = 12) drew or doodled with materials provided to them.

### 2.1. Participants

The sample was 12 children referred to receive DTGA for restorative dentistry and possible extraction of teeth. Patients were required to be eligible for GA, other eligibility criteria were: younger than age 10; classified based on the American Society of Anesthesiologists (ASA) as either ASA Class I (healthy) or ASA Class II (mild systemic disease); no disabilities affecting quality of life; and severe childhood caries. Patients with significant medical or developmentally compromised conditions and children younger than age 2 were excluded.

### 2.2. Process

Children received paper and crayons/pens for doodling/coloring during interviews with parents. Interviews lasted between 20 to 60 min. The first interviews, 1–2 weeks prior to DTGA, were to build rapport and establish familiarity to maximize the child’s potential to contribute to the research. Children were given blank sheets of paper and colored crayons/pencils, and invited to draw their perspectives on the dental experience using the prompt, “Please draw anything you can think of when you hear the word dentist or teeth.” A second, more specific prompt was, “Please draw your experience visiting the dental clinic to complete your dental treatment.” While children were encouraged to draw, they were not coerced to. The variation in number of drawings among children reflected this approach in collecting data that is important in qualitative study, allowing themes related to children’s experience of the DTGA to arise naturally from the data without bias or prompting.

Second interviews were conducted 2–4 weeks post-operative to seek child and parent narratives about the GA experience. We used the drawings as a stimulus for a dialogue with the parents and children about the children’s meaning making. All of the interviews were recorded and transcribed. We made field notes in each setting to capture contextual information about both the home and the dental contexts in which the children had produced their drawings. In addition, data collection involved a personal video diary, enabling child patients to document their thoughts and experiences related to DTGA as they went through the pre-, peri-, and post-operative pathway. Children were given a camera to video tape their experiences related to DTGA.

### 2.3. Analyses

Interpretation of children’s drawings evolved from the *Child Drawing: Hospital Manual*, an instrument designed to measure the emotional status of hospitalized school-age children [9,10] using both quantitative and qualitative components. It is a proven, validated instrument with good internal validity for measuring the emotional status of hospitalized school-aged children. The scoring of each drawing involved the use of the *CD:H Scoring Guide and Rating Scale* and the *CD:H Score Sheet*. According to the manual, the scoring of the drawing is divided into three sections. Part A has 14 items: position, action, length, width, and size of person; eyes and facial expression; color predominance; number of colors used; use of paper; placement on the paper; stroke quality; inclusion and size of hospital equipment; and developmental level. Each item is scored on a scale of 1–10, with 1 indicating lowest anxiety and 10 highest anxiety. Part B consists of eight items presumed to be pathological indices. The omission, exaggeration, and de-emphasis of a body part receive five points. Distortion or omission of two or more body parts, transparency, mixed profile, and shading receive 10 points. If the item is not present, a score of zero is recorded. Part C is a gestalt rating that calls for an overall response by the scorer to the child’s anxiety as expressed in the picture on a scale of 1–10 using the specific identifiers provided. A score of 1 indicates coping or low anxiety, and a score of 10 indicates disturbance or high anxiety. The total score on the *CD:H* is determined by adding the totals of Parts A, B, and C together. We were not able to score all drawings due to either child’s age or drawing’s context. In addition, the drawings were analyzed using Vygotsky postulations for context reading. In addition to social and cultural elements, the contexts in which children were asked to draw encompassed one-to-one interactions between the researcher and children, where children had some control over the nature of their engagement in data-generating activities.

## 3. Results

**Figure 1:**
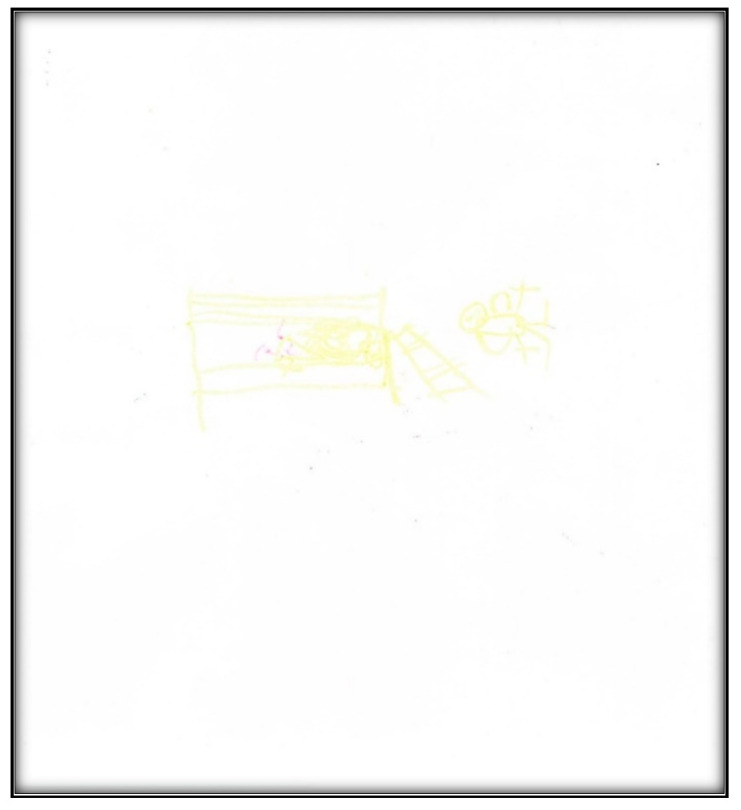
Child 1 Drawing 1 (pre-op).

Table 1 presents the demographic information of the child participants. There were eight girls and four boys, with an age range of 2.6 to 9.9 years, and a mean age of 6.1 (SD = 2.1). A sample scoring sheet for Figure 1 is presented in Table 2.

**Child 1’s drawings.** Child 1 drew herself sleeping in her bunk bed with the tooth fairy coming to fix her anterior “wiggly” tooth (Figure 1). A person lying in bed (a dependent position) portrays the child’s need for security or support. According to Clatworthy et al. (1999), placing a person in a defenseless position indicates anxiety and a sense of significance, coupled with loss of self and control of the environment [9]. The line drawn under the figure reflects the need to provide firm footing or grounding. Comparing the tooth fairy with the child, one notices there is a sense of life (motion) depicted in the tooth fairy, whereas the figure of the child is rigid; a rigid figure is often drawn by children who need to maintain control in situations where they feel small [15]. In addition, the figure lacks arms, portraying no life (in contrast, the tooth fairy has arms and wings, demonstrating action and life). Very anxious children sometimes draw bodies that have no apparent relationship to a head, and this is what Child 1 drew. The use of red indicates higher levels of anxiety. Child 1 used only two colors, although 12 colors were available. The basic premise is that children with increased anxiety do not have the energy to select many colors. The use of only a small portion of the paper, as in this drawing, is indicative of a very anxious child [16]. Likewise, light, fluffy line strokes indicate insecurity and a need for caution. The basic assumption is that all 5-year-old children will have reached the pictorial stage of development and could draw a picture that is reality-based; all children at this age should be able to draw a six-part person with head, eyes, mouth, body, arms, and legs [9]. The details of noses, ears, and hair are expected by age 7–8, although they are frequently found at an earlier age [9].

**Figure 2:**
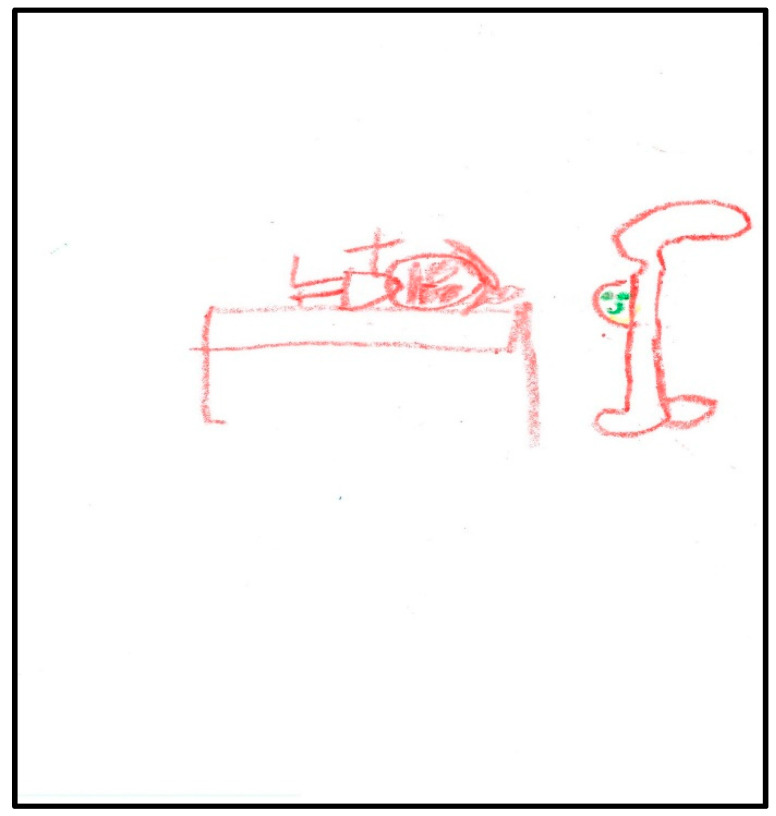
Child 1 Drawing 1 (post-op).

Child 1 drew four more pictures after the GA. The first draw depicts her in the surgery bed (Figure 2). Here there was presence of hospital equipment: the child drew the anesthesia unit (anesthesia machine), singling out the anesthesia mask. Hospital equipment indicates hospital anxiety; the items drawn often represent those that create the greatest concern. It is worth noting the picture was drawn only in red except for the mask which was green—the true color of the mask used. The child also put her name (removed to keep anonymity) on the drawing, using her first name as a signature (also in red). Drawn items often represent that which creates the greatest concern, and drawings are scored as they relate to the size of the person [9]. Compared to the child’s pre-operative drawing, the child here depicted herself in bed, but with an arm up, showing some evidence of life; at the same time, this was the arm used for the IV catheter during the GA (not depicted). The child projected her feelings by drawing a facial expression along with large eyes, indicating suspicion or hypervigilance. Disturbed children who sense they are being watched or controlled by others often draw large eyes with a skeptical view [17]. Interestingly, the child, here, drew a six-part person with head, eyes, mouth, body, arms (one arm only), and legs, consistent with development at age 5 (the drawing before GA lacked these details). Nose and hair are found, along with a hand and feet. The head is large, suggesting some preoccupation with the area, along with a pillow (used during GA) supporting her head and providing firm grounding. The total score given to this drawing was 94, indicating low stress.

**Figure 3:**
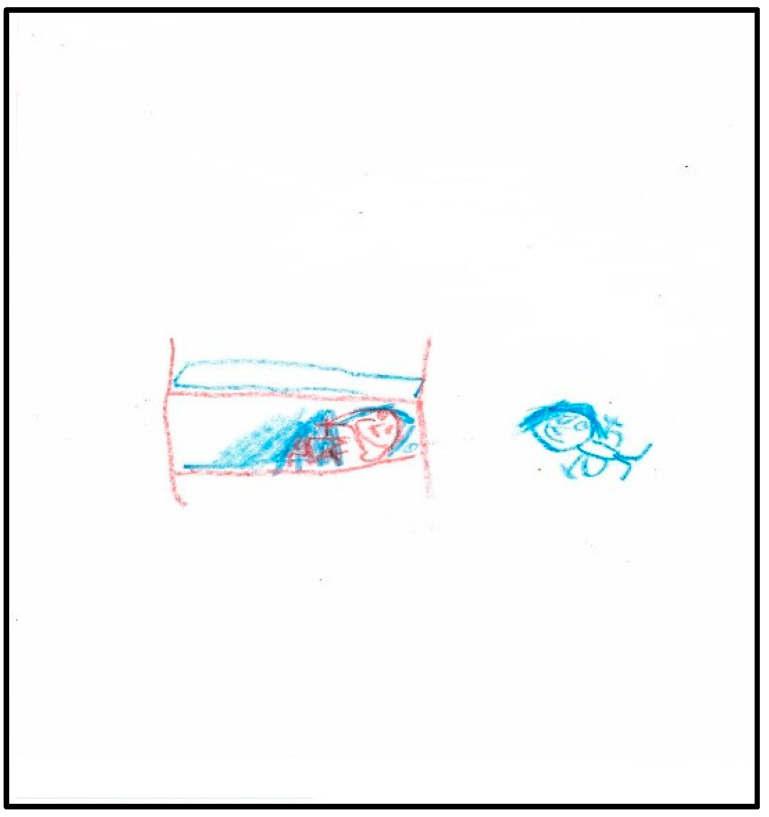
Child 1 Drawing 2 (post-op).

The third drawing shows the child in her bed at home after surgery with a tooth fairy next to her (Figure 3). In contrast to her pre-operative drawing, the tooth fairy here is colorful (in blue, generally a cheerful color) with a face with eyes, a nose, and a smiley mouth. The child’s head has eyes, a nose, and a smiley mouth, but the body is covered, yet present, under a blanket (transparency). According to Machover (1949), children in a normal developmental sequence of the human figure may show parts of the body through clothing until they develop a visual reality to their drawings (age 8–9) [18]. Transparencies before the age 9 are not considered pathological.

**Figure 4:**
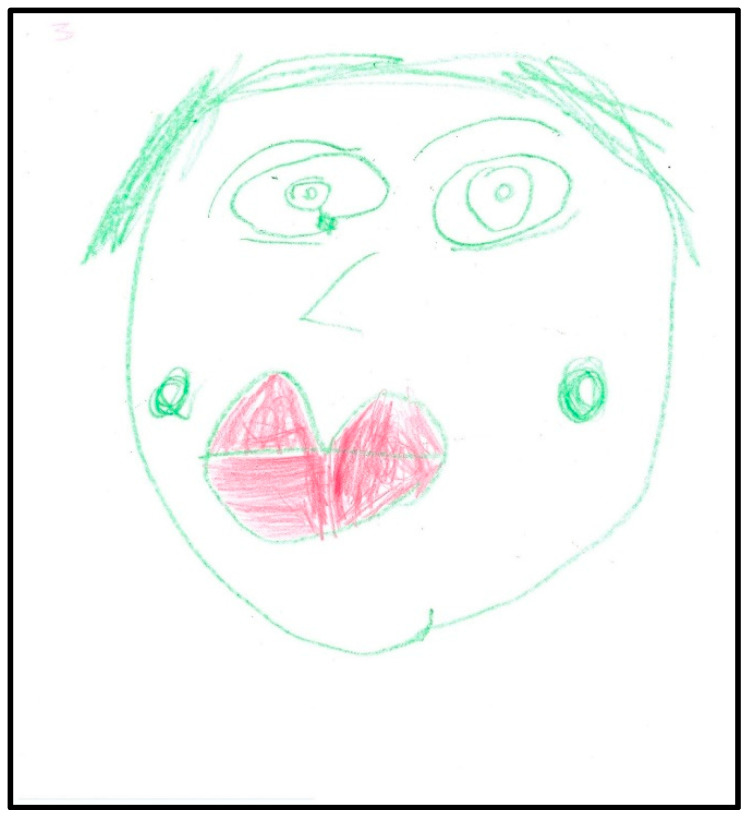
Child 1 Drawing 3 (post-op).

**Figure 5:**
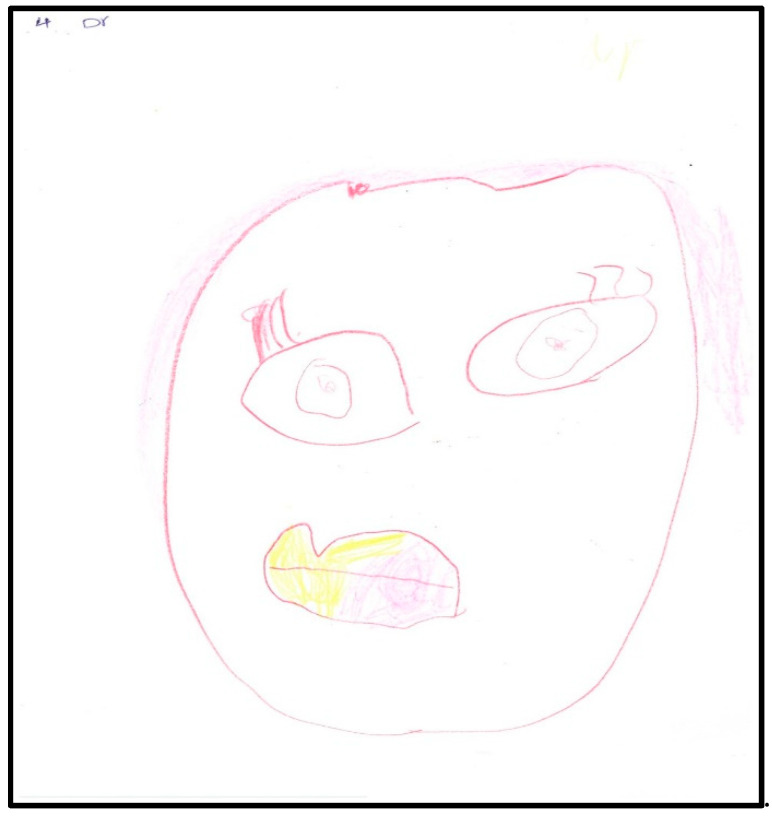
Child 1 Drawing 4 (post-op).

In two more drawings, the child drew her face (Figure 4) and her dentist’s face (Figure 5). During the interviews, the child indicated that she likes makeup (particularly eyeliner). The child used the whole sheet of paper, placing her drawing in the center of the paper (less anxiety). She drew large eyes with pupils and sclera, indicating suspicion or hypervigilance. In addition to eyeliner, the child drew a teardrop. The child shaded her mouth in red, indicating that the anxiety may be related to it. The dichotomy of health and sickness was represented by contrasting the child’s mouth to the dentist’s mouth, with the latter lightly shaded in yellow and pink, generally considered cheerful or happy colors. In these drawings, the child used almost all the space available on the paper, placing the faces in the center. Children who are less anxious tend to distribute contents over the whole of the paper and draw in the center.

**Figure 6:**
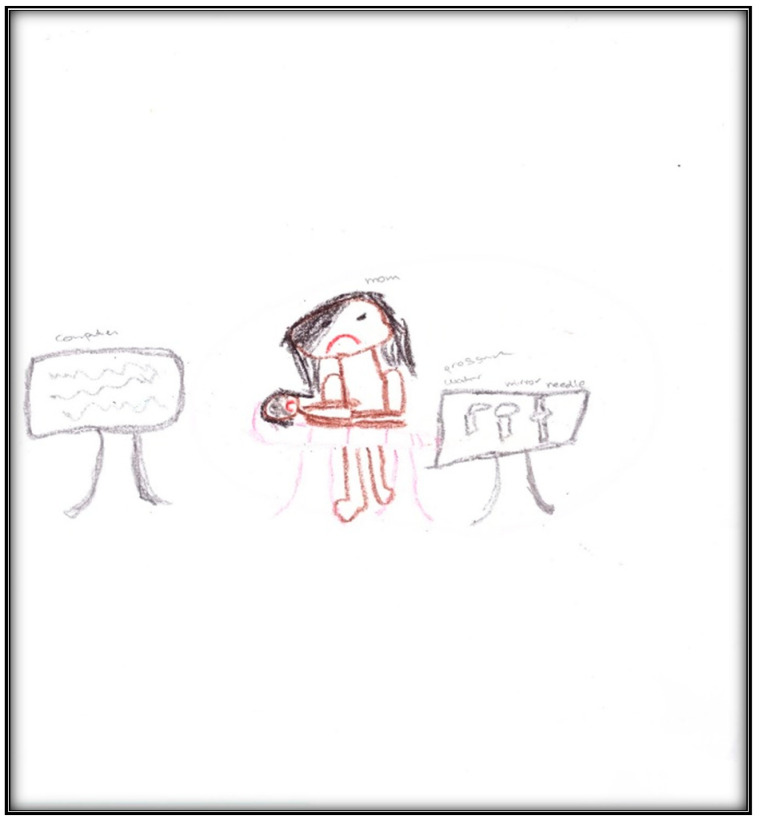
Child 2 Drawing 1 (pre-op).

**Child 2’s drawings.** The child’s first pre-operative drawing (Figure 6) depicts the mother’s description, in which we see a needle, a dental mirror, and a dental air–water syringe, all in black, considered to indicate higher levels of anxiety. The screen (that the mother referred to) was also present in the child’s drawing, again in black. The child placed herself in a defenseless position in the dental chair, crying, with a frown drawn on her face, which is also the mother’s facial expression. The child used three colors (brown, red, and black) indicating an intense sense of threat, fear, and loss of control frequent with increased anxiety. The child used a relatively small portion of the paper, using the left area, suggesting an orientation in the past. Importantly, and in contrast with Child 1, transparency (showing part of the body through clothing or objects (dental chair)) is not found. The scoring given to this drawing was 138, indicating an above-average stress level.

**Figure 7:**
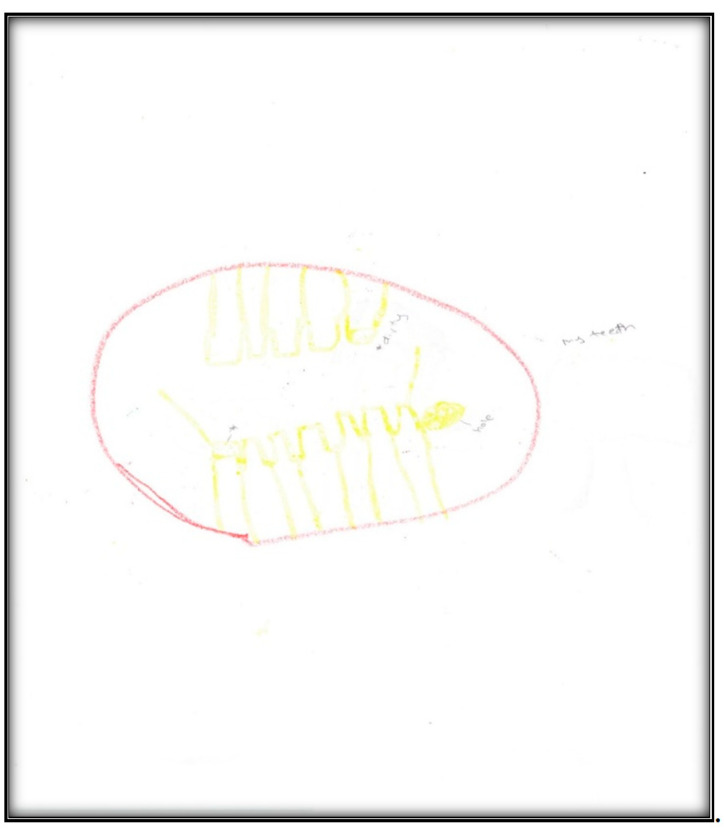
Child 2 Drawing 2 (pre-op).

In the second pre-operative drawing (Figure 7) the child shows her teeth, depicting dirt on both sides of her mouth and a hole on one side, consistent with her description that, “Like, when I am chewing, this side hurts, but when I am chewing on that side, doesn’t”.

The categories identified from this drawing in terms of oral health are the dichotomy of health and sickness, with some teeth appearing straight and healthy, while others appear with “dirt” and a “hole”. Her level of abstraction allowed her to associate unhealthy teeth with imperfections. This also reflects that the child perceives oral health as a process.

**Figure 8:**
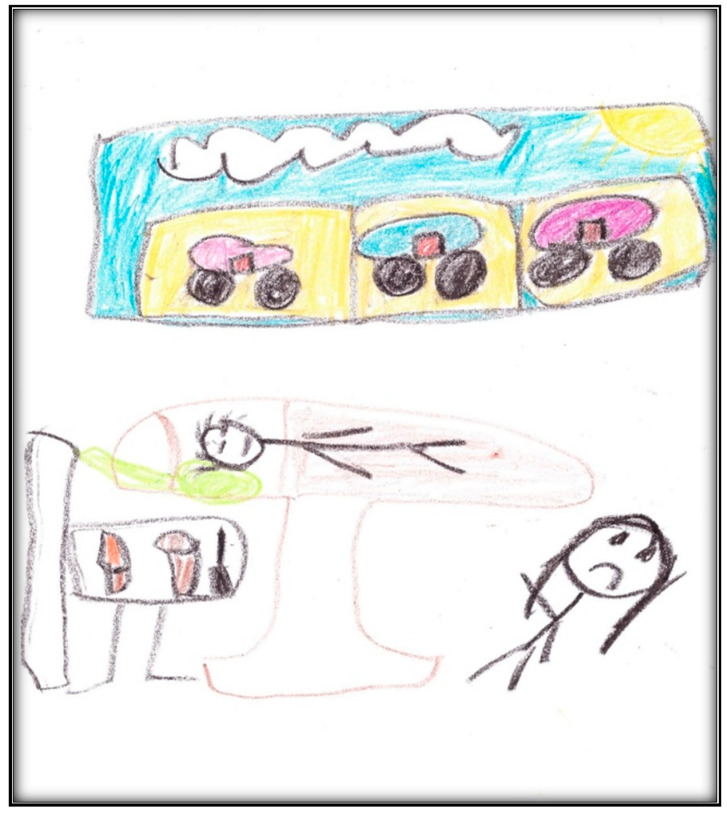
Child 2 Drawing 1 (post-op).

The child drew two informative drawings post-operatively. The first is herself, her mother, dental instruments (needle, air–water syringe, the mirror), and the GA machine (Figure 8). The child is in a state of submission, indicating she has no choice but to go to “sleep”. The mother, next to her surgical bed, is frowning, indicating negative emotions. In contrast, the child’s lack of facial expression may indicate an emotional disturbance or an attempt to mask her true response to the situation. These representations of the mother and child are consistent with the statement made by the mother:

“When they had called her in and I went with her behind the doors, there, they got her to blow a balloon, and I was, like, holding her hands crying because she is my baby, so [laughing loudly]—So she is my last child. I was scared. Really scared.”

The child stated: “I was pretty scared, but for just a little time, then I calm”. At another point, she said:

“I was a little bit scared and a little bit shy because—Because I had to put that thing on, and I was, like…had no friends, but a kid was there [all laughing] and these doctors gave me that tasty thing that I don’t like…I don’t know what they did, but they said ‘open your mouth’ and I did. They had to check how much temperature was, but I didn’t know because I didn’t know.”

In the drawing, we see a green surgical mask next to the child’s head (it is the most prominent part of the GA machine). The child said that she loves green, as well as the “balloon” (i.e., the surgical mask), in contrast to the needles. She said

“I kind of liked the balloon. I stared at it a little bit, but they told me to breathe in and that was, kind of, scared because I thought they were gonna, like, put needles in my gum because last time they did it and it hurt it. It was really bad, and I did not like it.”

According to the child, this drawing represents the operating room (although the term used by the child was “the thingy”, “I drew me and the thingy…I don’t know what is called”), and, as it appears in the drawing, it contains a big window showing a blue sky, yellow sun, and white clouds. A parking lot along with some cars can also be seen through the OR window. This shows that the child could see beyond the OR building, ensuring expansion of the field of vision and the mobility needed to relate to the closed environment that is limited because the child is in bed.

In contrast to the pre-operative drawing, the child used almost all the space available on the paper, signing her name below the drawing at the bottom center. She also chose to use more colors (nine colors); children who feel better about themselves in the hospital are more likely to have many colors in pictures [9]

Compared to the pre-operative drawing, the only regressive items are stick figures drawn to represent Child 2 and her mother. Stick figures are often drawn by children hesitant to reveal themselves. Although the figures are sticks, with no clothing (figures without clothing relate to the child’s sense of exposure), the heads are disproportionately large and have distinct facial expressions (particularly the mother, who seems extremely worried and unhappy, whereas the child’s face lacks facial expression). The score given to this drawing was 84, indicating average stress.

**Figure 9:**
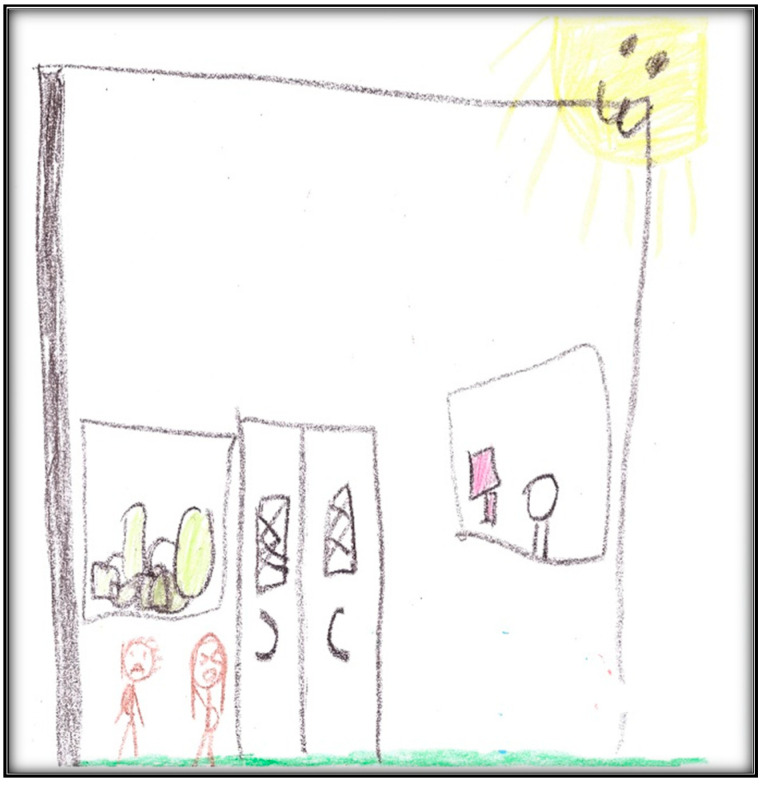
Child 2 Drawing 2 (post-op).

Child 2’s last drawing shows the waiting area of the surgical center (Figure 9). The drawing depicts two doors, one for the dental surgical room (i.e., dental OR) and the second for non-dental medical procedures. It includes the learning station along the wall, which is an educational space for children.

Two children can be seen in this drawing: Child 2 and another child who seems to be making fun of her. During the interview, the child described how bullying at school and other children making fun of her teeth affected her perceptions in the waiting area.

In this drawing, we can see green furniture—the child said she likes green—and the sun the child described as “my cute sun!”

**Figure 10:**
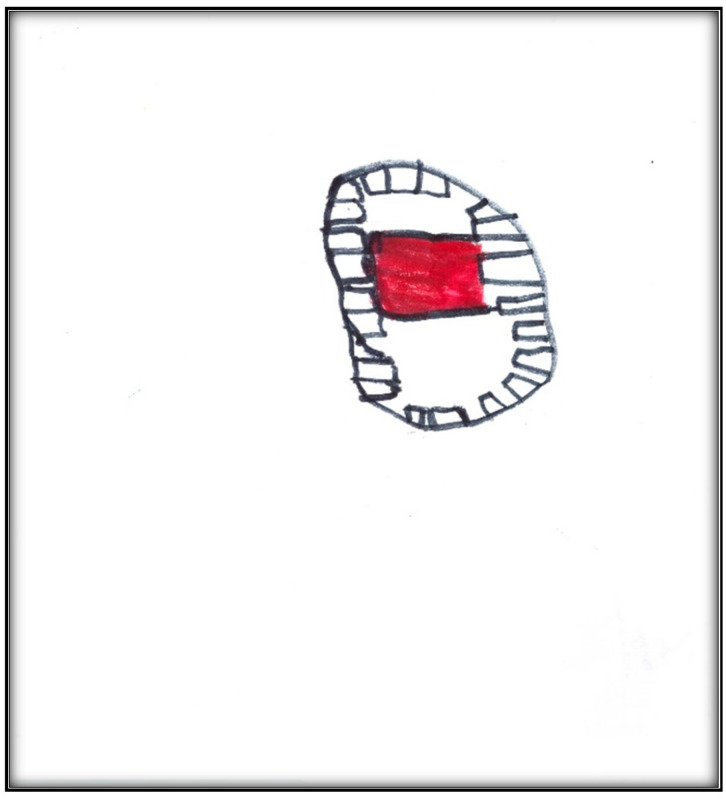
Child 3 Drawing 1 (pre-op).

**Figure 11:**
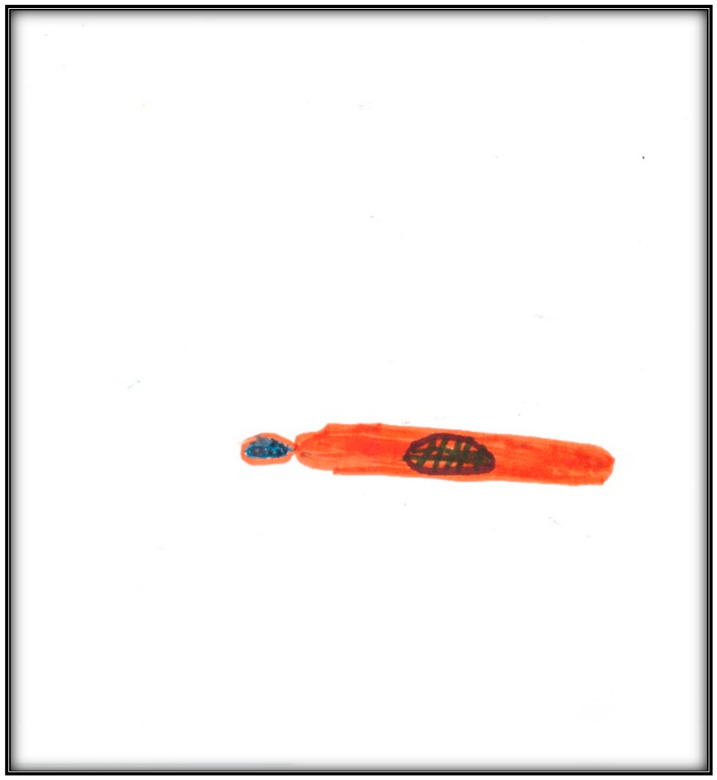
Child 3 Drawing 2 (pre-op).

**Child 3’s drawings.** During the pre-operative interview, the child made two drawings. The first depicts her mouth, including teeth and tongue (Figure 10). There are spaces representing lost teeth and a tooth that seems broken and hurting. The dichotomy of health/sickness is well represented in this drawing. The second drawing depicts a toothbrush (Figure 11). When the child was asked why she drew a toothbrush, she replied: “It helps you take care of your teeth more”. Although the mother told the child what to expect the next day during the dental surgery, the child explained as follows: “They put, kind of, something inside my mouth and they will ask me to blow up a balloon. Then I will fall asleep and then they can do the stuff they need to”. The child did not draw anything directly related to the upcoming procedure.

**Figure 12:**
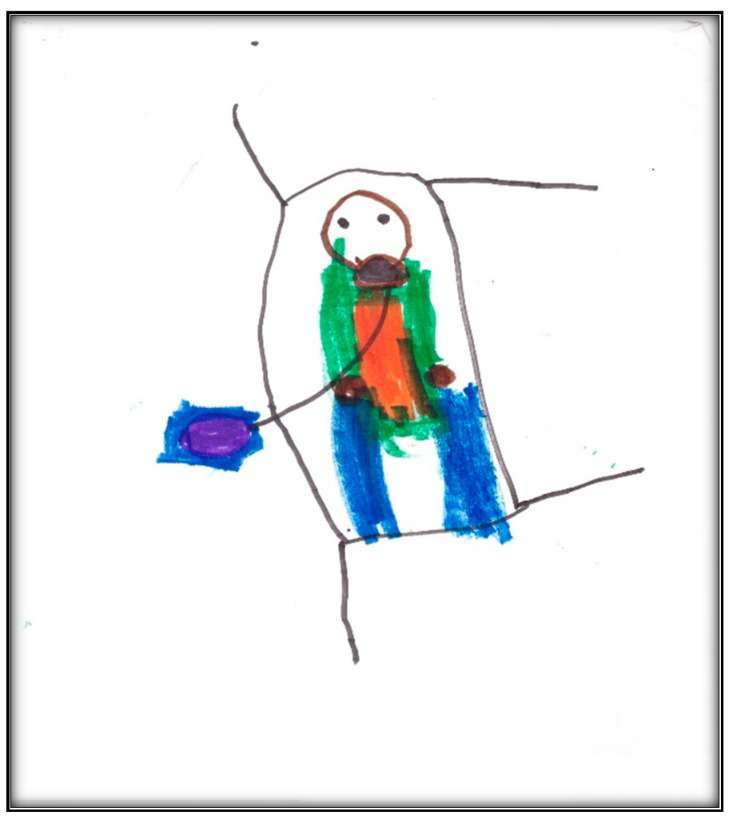
Child 3 Drawing 1 (post-op).

The post-operative drawing, however, depicts the GA induction as perceived by Child 3. We see the mask (in black) and the balloon (in purple) the child blew into, in addition to the green and orange scrubs the child chose to wear (Figure 12). The child described herself as “scared”, “nervous”, and “I didn’t want to go to sleep”. Contrasting the pre-operative drawings with this drawing completed post-operatively, one notices the focus shifting from the dental conditions, represented by the drawing of the mouth and a toothbrush, to the general wellbeing condition, represented by the child in the surgical bed with a surgical mask over the lower part of her face. The patient is the only person depicted in the picture. The figure here seems rigid and in a defenseless position, coupling a loss of self and control of the environment. The shading in this drawing indicates generalized overall anxiety. The overall affect of the drawing can be described as disturbed, overwhelmed, sad, and defeated. The total score for this drawing was 117, indicating average stress.

**Figure 13:**
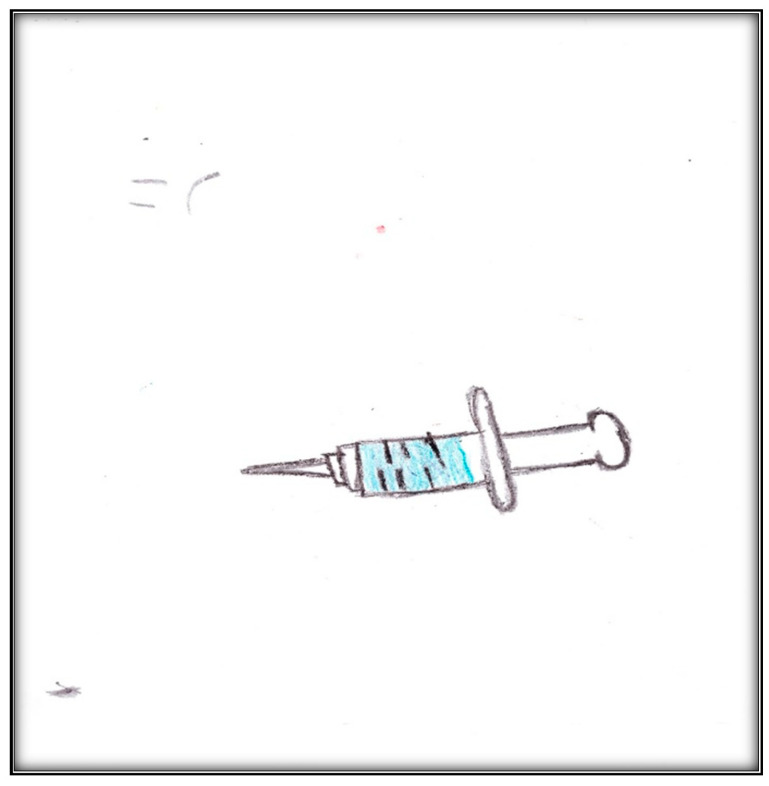
Child 4 Drawing 1 (pre-op).

**Child 4’s drawings.** The girl drew two pictures pre-operatively. The first represented the tool she was most afraid of: the needle (Figure 13). During interviews, the mother revealed that she too feared needles: “I would be hating needles”. The child drew a supersized syringe. The picture depicts a ready-to-use syringe, including the medicine to be injected (in blue) and a sharp needle. She also drew her sad face, indicating negative feelings in a threatening situation. The face is small compared to the syringe and sheet of paper; children who are threatened and overwhelmed are likely to draw small people in large environments [19].

**Figure 14:**
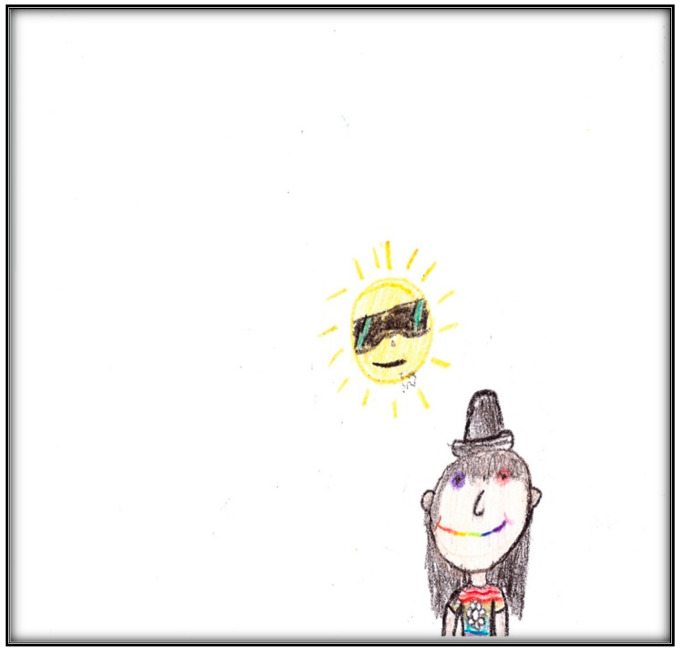
Child 4 Drawing 2 (pre-op).

The second pre-operative drawing (Figure 14) is bright, happy, and confident. Child 4 has a smiley face and a cheerful sun. Note the use of black as the dominant color and the small space used. Drawing on the right side indicates less anxiety and a sense of hope. Although the child carefully drew many details, she drew her mouth as a smile, rather than an oral cavity. This might mean that her teeth hurt her when she breathes and maybe because they are yellowed.

**Figure 15:**
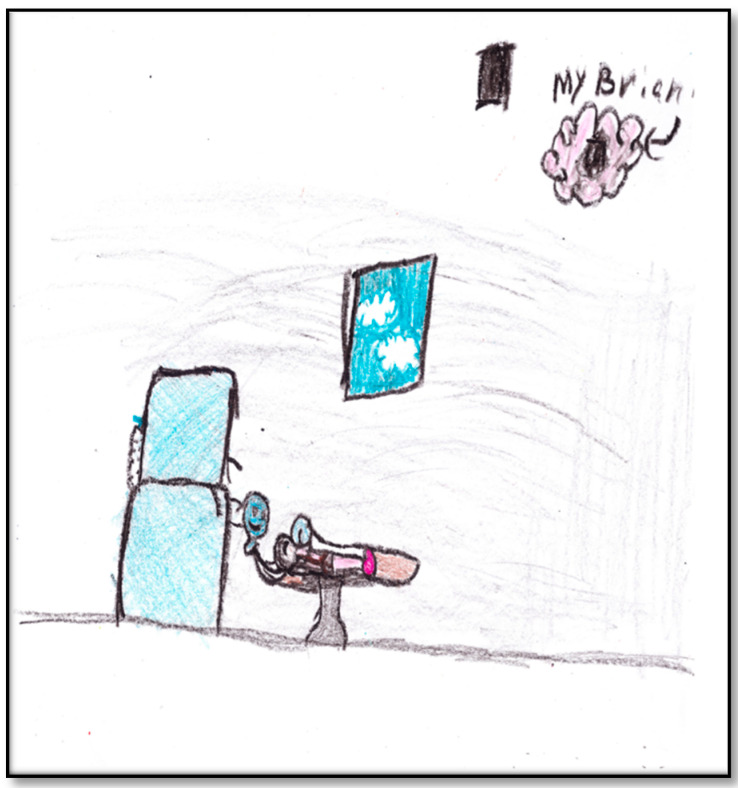
Child 4 Drawing 3 (pre-op).

**Figure 16:**
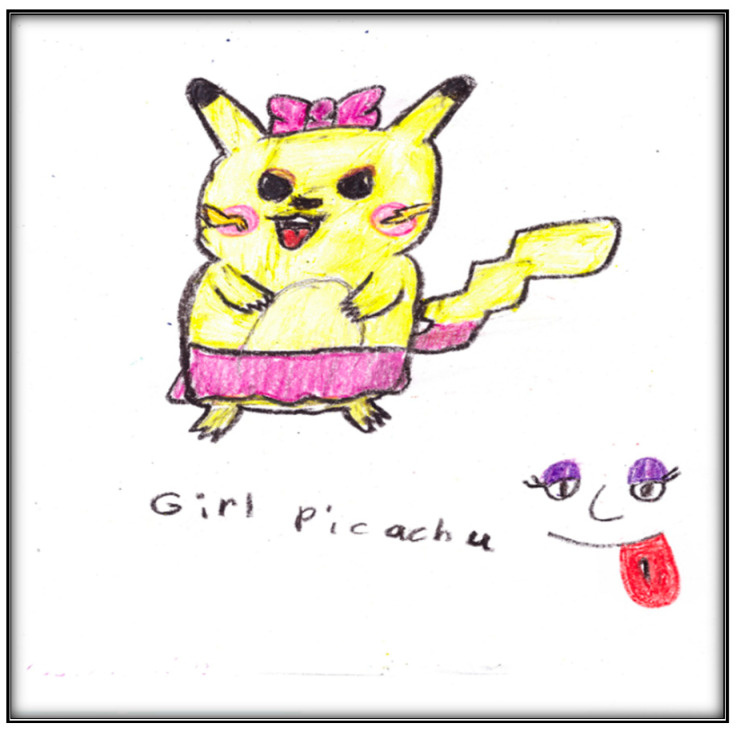
Child 4 Drawing 4 (pre-op).

**Figure 17:**
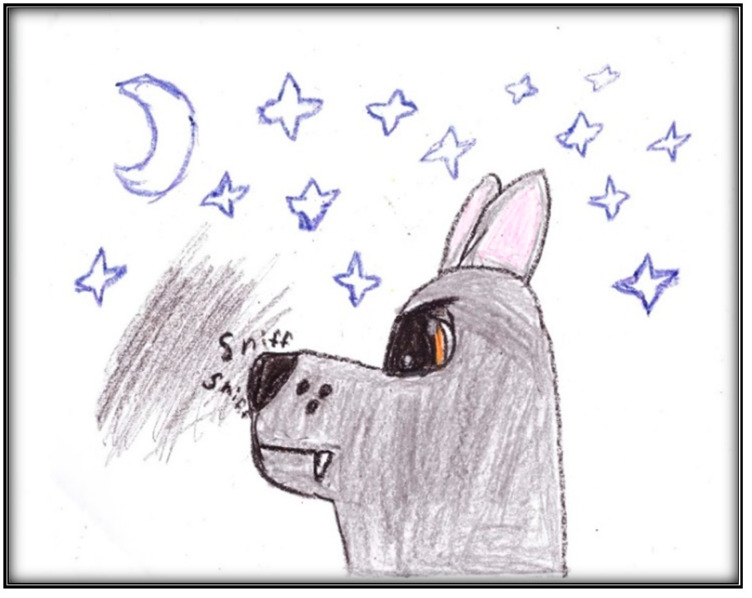
Child 4- Drawing 5 (pre-op).

During the post-operative interview, Child 4 made three drawings (Figure 15, Figure 16 and Figure 17); two depict the Pokémon characters of a dog and a “Girl Picachu”. (The character’s name is “Pikachu”). When was asked about the drawings, she related a bad dream she had after the surgery.

The third drawing by Child 4, directly related to her GA experience, depicts her in OR with the GA machine dominating the scene (Figure 15). The child depicts the surgical mask as a smiley face (“because it is the one that it keeps how I breathe and…my heartbeat”; the heart was pink). The child drew the only window in the OR with a couple of clouds showing through. Most importantly, perhaps, is what the child drew at the upper right corner of the paper: it was her “brian” (i.e., brain) that is now “blank”. As the child stated: “Now, I do not dream anything”. Shading is seen everywhere in this drawing. Shading is always related to anxiety. The pattern of shading in this drawing refers to generalized overall anxiety, and coloring is in all areas, including the sky, ground, and background. Further, black was used more extensively than any other colors in both drawing lines and shading. In Western culture, black is characteristic of intense anger, aggression, threat, fear, or loss of control frequently found with increased anxiety [9]. The child drew a closed door in black to represent her blank brain. She drew the door twice, once in the picture representing her brain and another one just close to the brain, cluing us to the emphasis she places on her brain as important to her or concerning her emotionally. Interestingly, the child signed her drawing at the right bottom of the paper, also in black. When the child was asked what she saw in her dreams before her brain went blank, she replied: “Unicorn, wolves, and wilderness…and broken-down car”. Another important note: although this child felt the pain of the IV cannula in her arm, she did not draw it.

**Figure 18:**
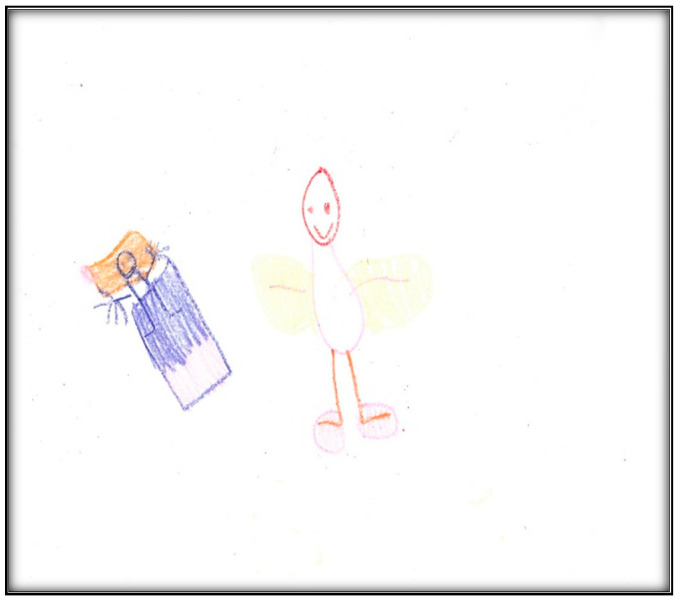
Child 5 Drawing 1 (pre-op).

**Figure 19:**
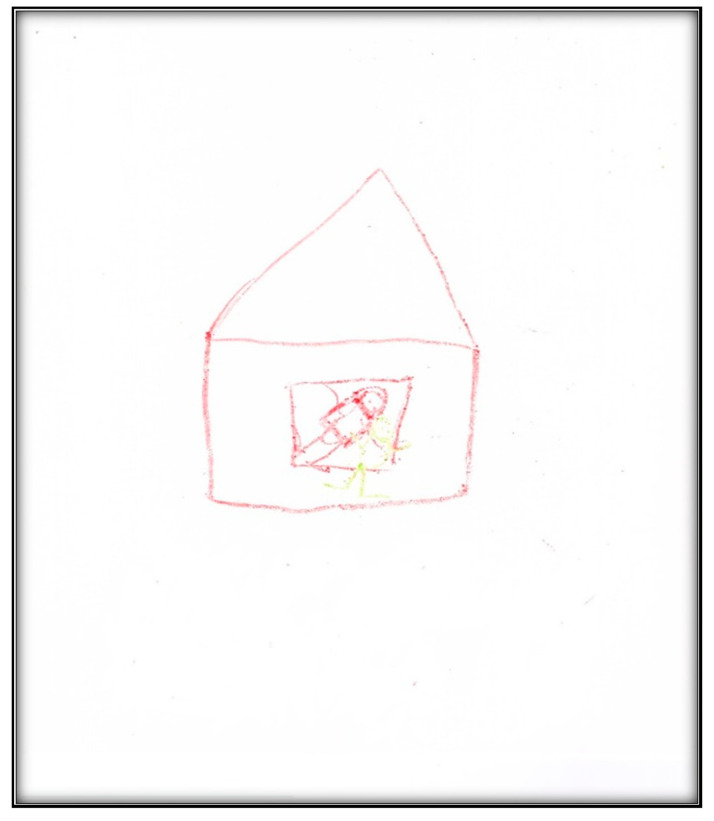
Child 5 Drawing 2 (pre-op).

**Figure 20:**
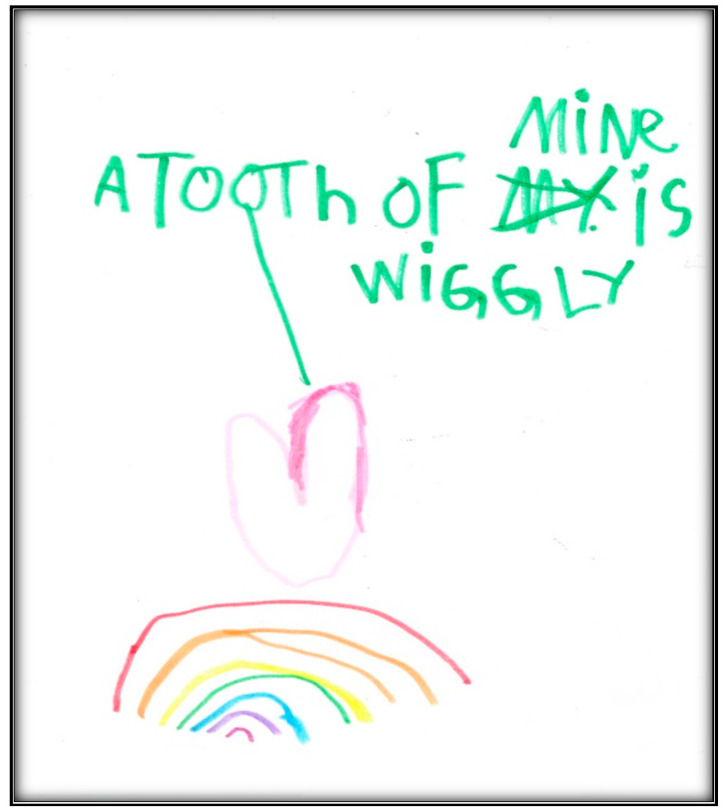
Child 5 Drawing 3 (pre-op).

**Figure 21:**
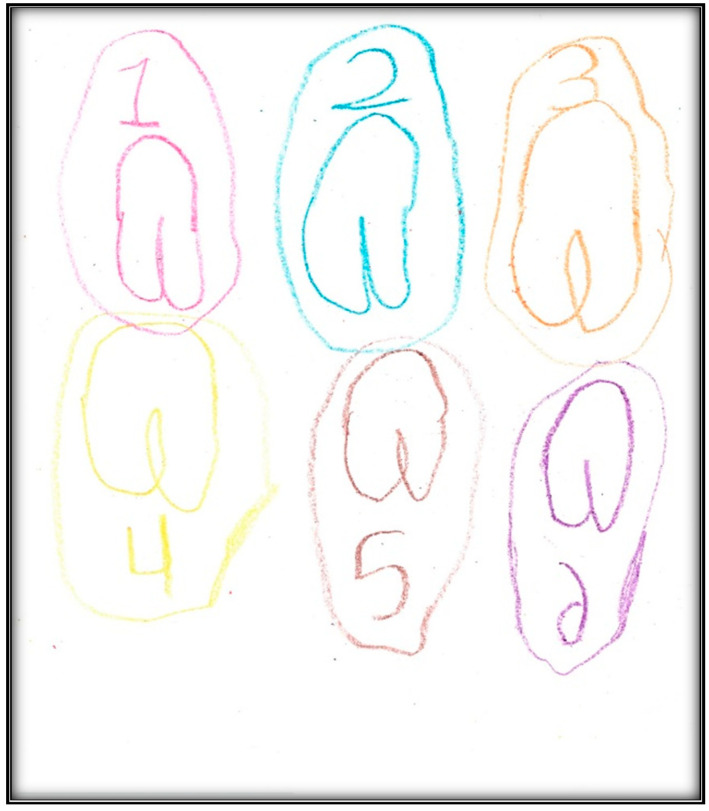
Child 5 Drawing 4 (pre-op).

**Child 5’s drawings.** The child drew four drawings during the pre-operative interviews (Figure 18, Figure 19, Figure 20 and Figure 21). All were related to her dental issues. Two drawings had the tooth fairy as the main theme (Figure 18 and Figure 19). In her first drawing, the child depicted herself sleeping at home with a tooth underneath her pillow and a tooth fairy visiting, hoping replacing the lost tooth with a small payment (as the child explained during the interview). In this drawing, we see part of the child is apparent through the blanket, which is drawn in two colors. Transparency is a normal development sequence until children develop a visual reality to drawings, which generally occurs around age 8–9. Also of interest is that the child drew five fingers on her hands and her legs have feet, which is an advanced developmental level in drawings at this age. There is also a basic Tinkerbell-type tooth fairy with wings and a smiley face. The facial expression of the child seems neutral in contrast.

The family was visited the day before the dental surgery to give the video camera to the child. When we asked the mother how she had prepared her child for the next day’s appointment, she explained that she had prepared the child for seeing a different dentist and the fact that there would be medicine allowing her to sit still for a long time.

**Figure 22:**
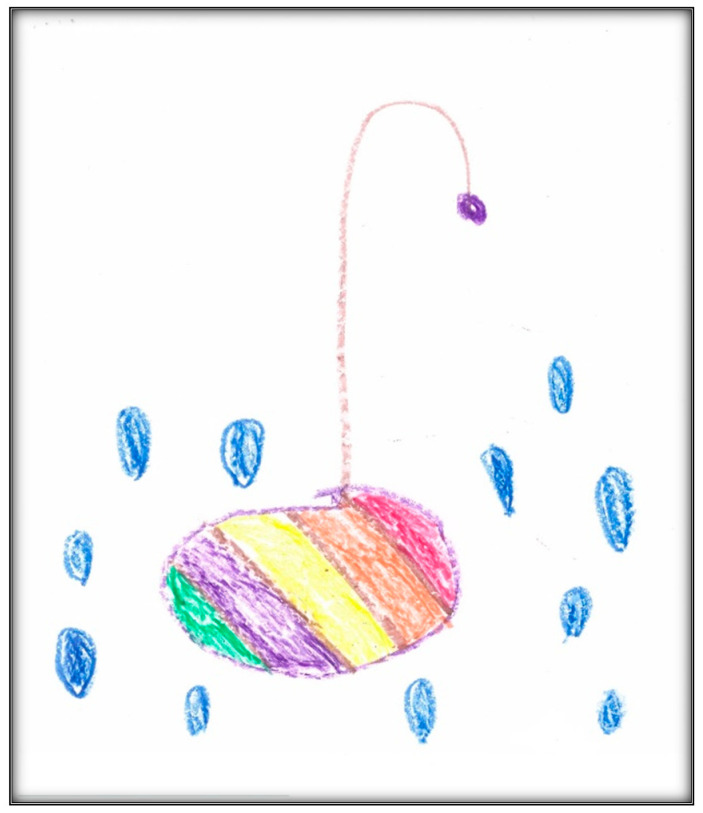
Child 5 Drawing 1 (peri-op).

Family 5 was visited at home the day after the dental surgery for a peri-operative interview with mother and child. The child drew two pictures. The first depicts an umbrella with six of the rainbow colors (Figure 22). You can see raindrops in the seventh rainbow color, the blue. This drawing can be interpreted from two perspectives. In one, the picture is as a way of denying the hospital experience from the day before. This argument is supported by the conversation between the mother and child at the beginning of the interview:

Q: (to child) “So would [you] come here and draw something for me related to your visit yesterday?” (Mother 5: “So something to do with your visit yesterday to the hospital.”)

Child 5: “I do not know what you mean!” (Mother 5: “Draw something that comes to your mind about yesterday, your experience, and your appointment at the hospital yesterday. So you can draw, like, a picture of the operating room or draw a picture drawing your pajamas (laughing).”) Child 5: “Okay.”

**Figure 23:**
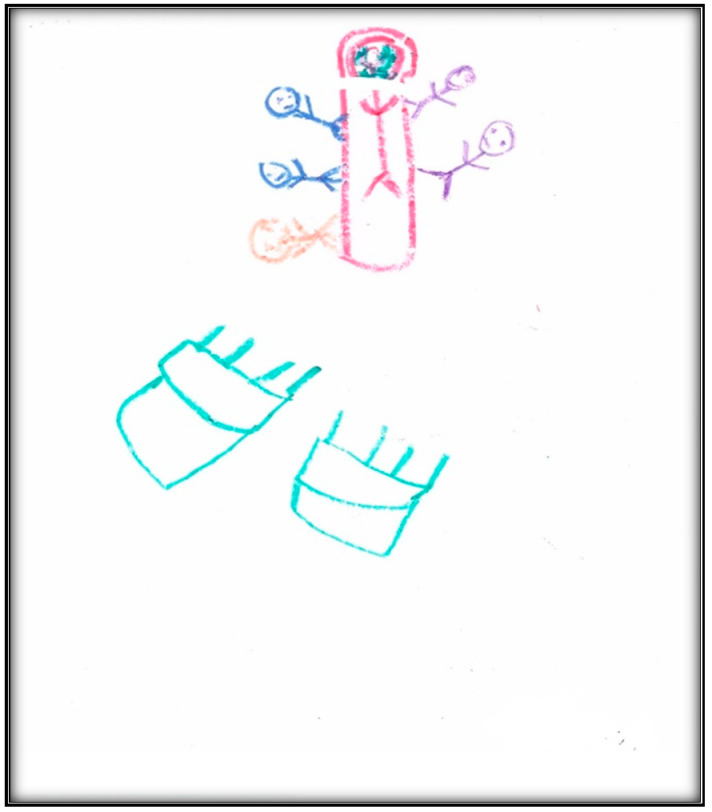
Child 5 Drawing 2 (peri-op).

From another perspective, the mother told us that the child liked umbrellas, and to have a rainbow you need some rain. It is noteworthy that Child 5 chose to draw a non-dental, non-medical situation. Taylor, Roth, and Mayberry (1976) suggested that some anxious children do not draw pictures of the anxiety-provoking situation [6]. A drawing unrelated to an experience might represent simply something else a child was thinking about, or it may reveal a high level of anxiety preventing a child from thinking about the dental/medical experience. After repeating the instructions regarding the drawing, the child drew another relevant picture, placing images low on a page which may indicate insecurity. In this drawing, Child 5 depicted herself lying in OR, with a mask in green covering her face and four medical personnel, in addition to her mother, surrounding her. You can see two vacant chairs in the room away from the bed (Figure 23). This operating room is different from other children’s representations of the room; there are no windows, and the mother is present (the anesthetist allowed the mother in). When asked if she had anything to add, he Mother 5 said:

“Umm…umm…I do not think so. It was good experience and we really like that doctor. She was very…explained things nice and very patient, sweet and kind and…umm…the two anesthesiologists that were there, like, one doctor and a resident, the two explained things nice. They were talking and singing songs from Frozen and having fun with her.”

The total score given to this drawing was 108, indicating average stress.

The final interview with Family 5 was conducted 4 weeks post-DTGA. During this interview, it was evident that Child 5 was herself again compared to the visit 2 days after surgery.

**Figure 24:**
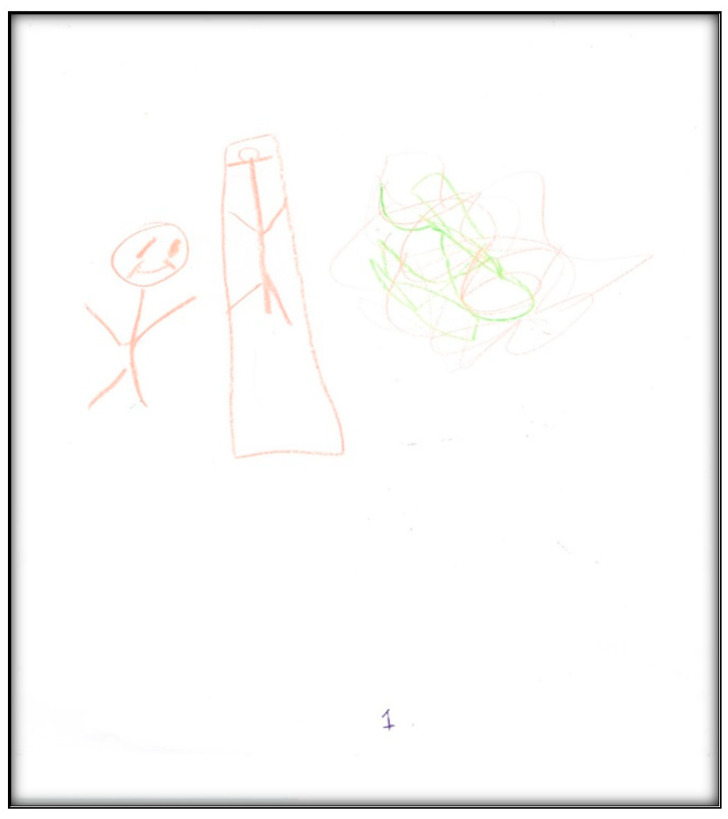
Child 5 post-operative drawing.

**Figure 25:**
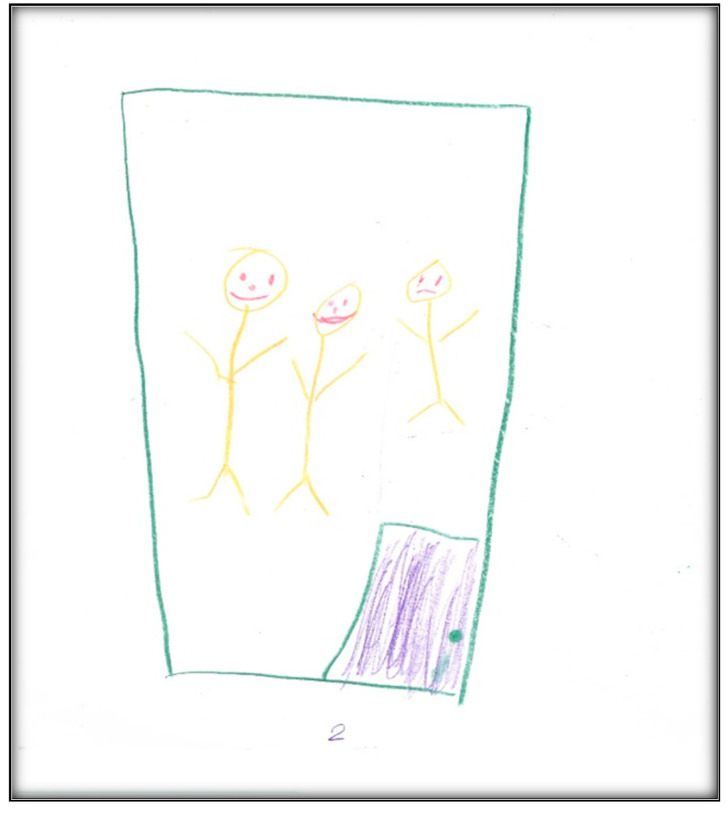
Child 5 post-operative drawing.

**Figure 26:**
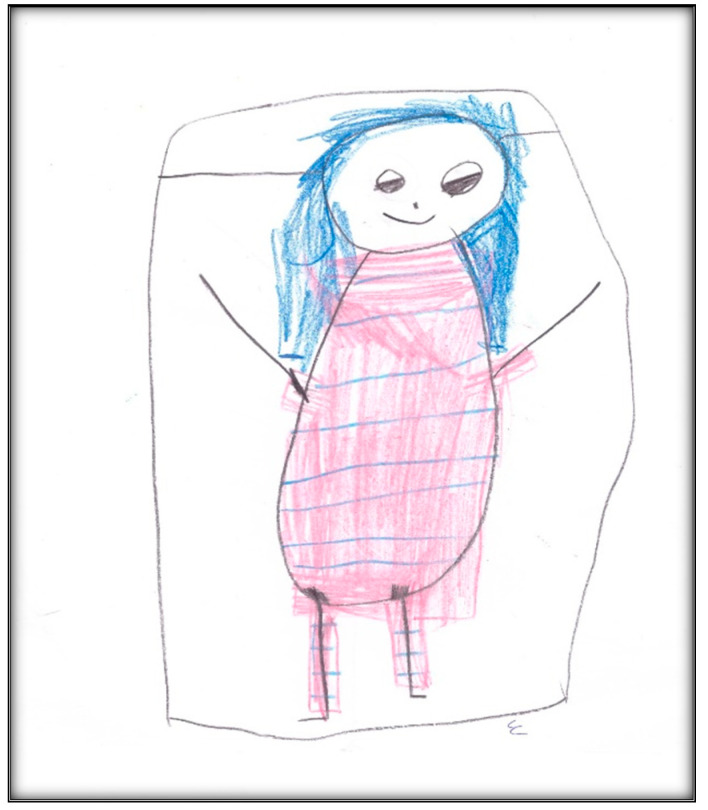
Child 5 Drawing 3 (post-op).

**Figure 27:**
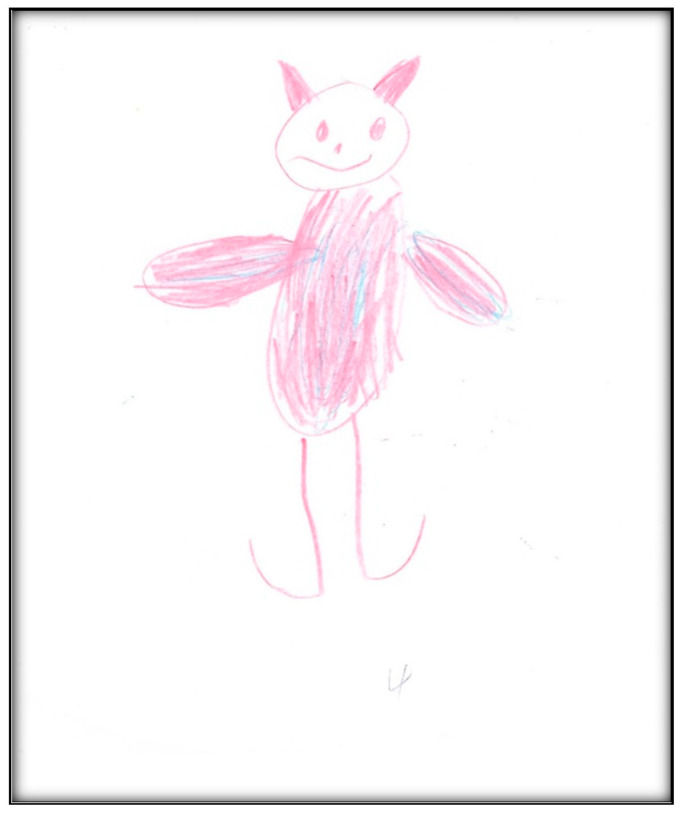
Child 5 Drawing 4 (post-op).

**Figure 28:**
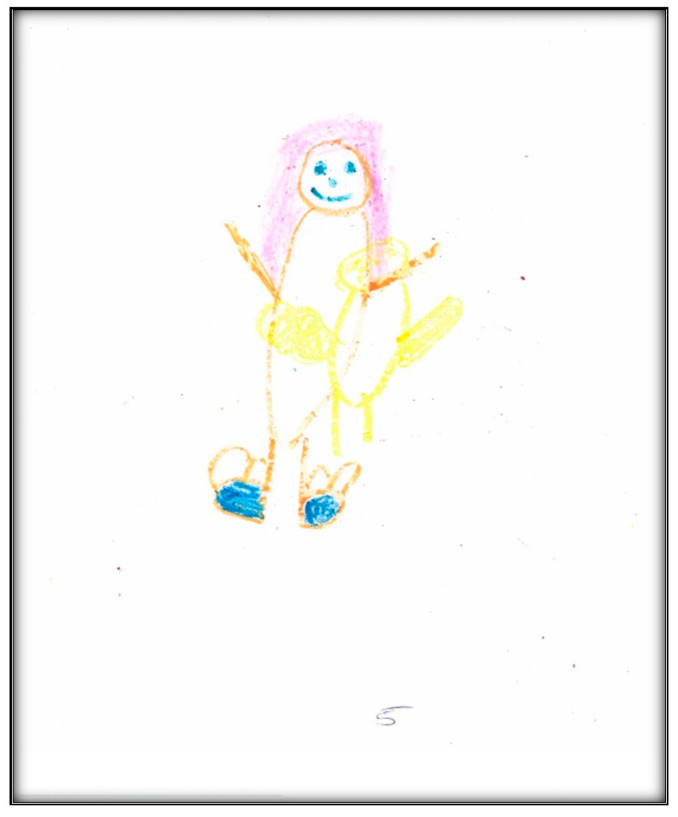
Child 5 Drawing 5 (post-op).

**Figure 29:**
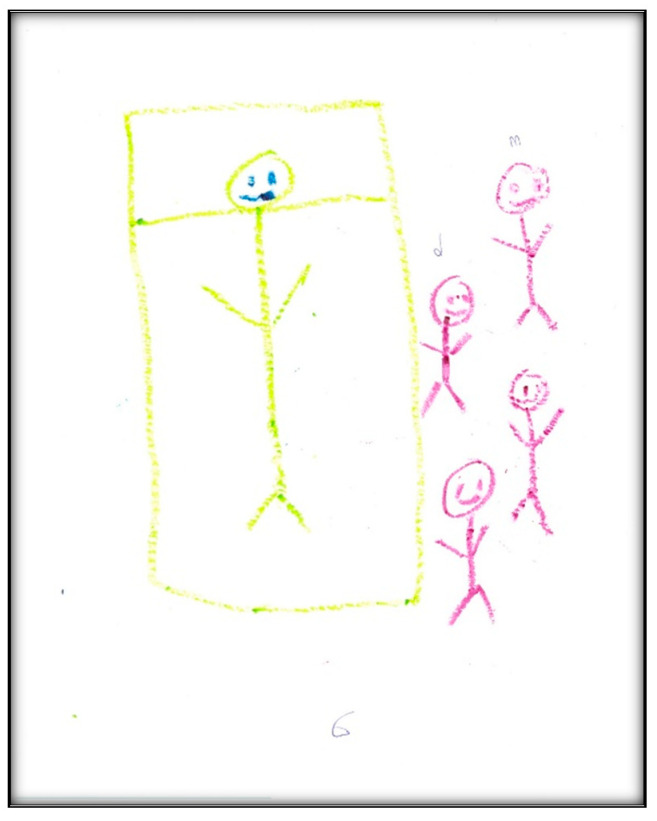
Child 5 Drawing 6 (post-op).

**Figure 30:**
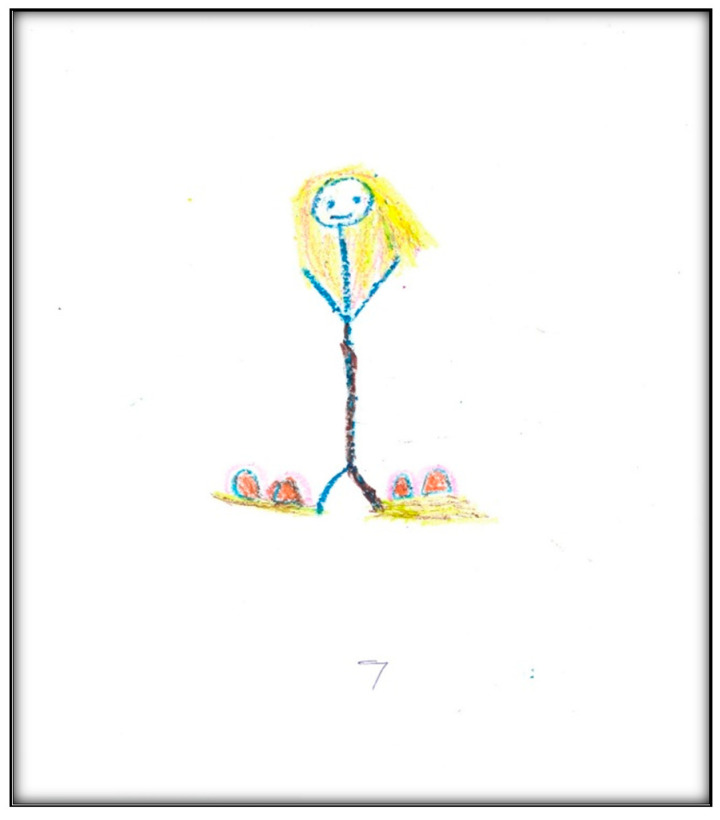
Child 5 Drawing 7 (post-op).

**Figure 31:**
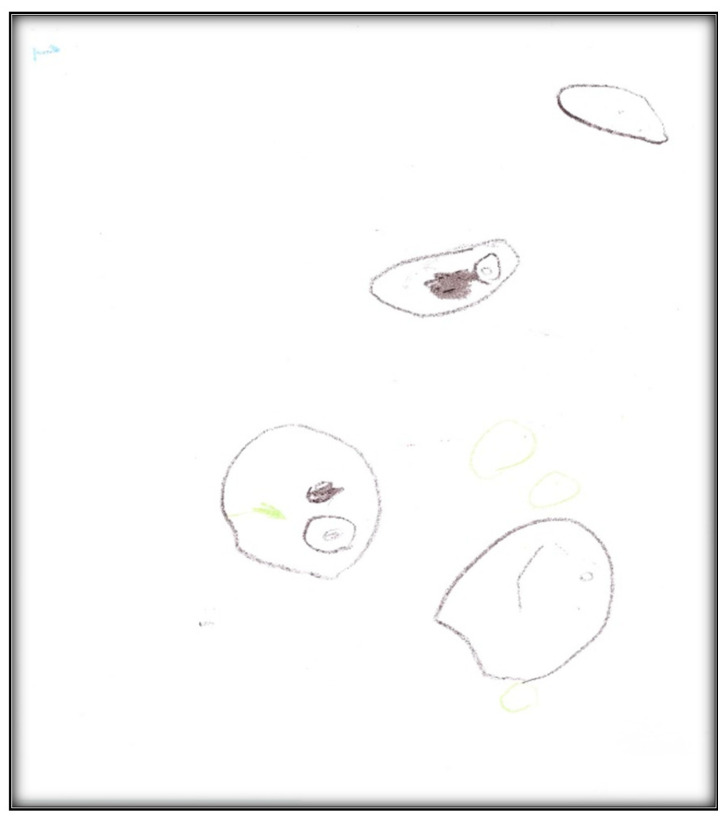
Child 7 Drawing 1 (pre-op).

During the final visit, the child held a teddy bear given to her in the hospital, except when she was drawing. Unlike the previous visit, where the child was repeatedly asked to draw something related to her experience at the hospital, she was internally motivated to draw and talk openly about her experience. She completed several drawings (Figure 24, Figure 25, Figure 26, Figure 27, Figure 28, Figure 29, Figure 30 and Figure 31), all addressing her medical/dental experience at the hospital. Child 5’s father also briefly joined this discussion.

In the first post-operative drawing (Figure 24), there is a child lying in the OR with her head on a pillow and a smiling female dentist next to her bed. The cords to hook up the OR equipment are depicted in two colors. The child placed her first drawing near the top of the page; placement high on the page may indicate optimism and striving to reach goals, or the drawer may be using fantasy to achieve goals [20,21]. Although the child drew stick figures and the figure depicting the child has a small-sized head with no facial expression that provides little information, she drew a large-sized dentist’s head with a distinct smile. The child confirmed the figure was a female dentist, not a doctor, and the dentist is smiling. Another item to note is that Child 5 opted to use two colors, red the dominant; both elements are more characteristic of intense negative feelings.

The second post-operative drawing (Figure 25) shows parents talking to a dentist at her office. The drawing has elements of interaction that suggests the dynamics of the personalities. The dentist is clearly smiling, as was confirmed by Child 5. The mother seems less happy than the dentist, and the father is frowning. The interview conducted 2 days after surgery explains this representation. The mother mentioned “we were a little bit nervous because we felt if there is to be any complication it would be from having general anesthetic. I think that was—A kind of scary factor there”.

Another note from this drawing is related to the size of the figures: the larger figures are the more dominant the personalities (the dentist in this drawing, while the father seems short and at some distance from the dentist and mother). Finally, the child does not depict herself in this drawing, as if she is observing the dynamics between the dentist on one side and her parents on the other.

A third drawing by Child 5 depicts herself in bed wearing pajamas (i.e., surgical scrubs), drawn in red and white (Figure 26). She added more detail to her head, including sleeping eyes, a smile, and hair. There is also a pillow below her head, a constant in her drawings. The smile, along with the closed eyes in this threatening position, may indicate the need to deny what happened or be an attempt to mask her true feelings about the situation and avoid contact with the outside world. The large eyes shown here, compared to child’s other drawings where her eyes were drawn as pinpoints, may indicate suspicion or hypervigilance. Although the child in the drawing is asleep, her arms are up, indicating a sense of life.

The fourth drawing (Figure 27) depicts a teddy bear the child received. We know that the actual teddy bear is white, but she drew it red and blue. In another drawing, the child drew herself on a bed and holding the bear (Figure 28). Both mother and child appreciated the bear as a positive thing during a stressful event. Mother 5 said:

“The way that the doctor and the anesthesiologist—that they came in in the holding room before surgery…and explained everything….. That was really good…. I think they treated her very well. I think that was really good she got that teddy bear there.”

In Figure 29, Child 5 again depicts herself in a hospital bed with three “doctors dentist” in addition to her mother next to her bed.

In the last drawing, Figure 30, you see Child 5 and her extracted teeth off to the side. In the interview when we asked about her teeth, she replied, “My teeth are NOT white all the way in the computer as they look like now because they were a little bit fuzzy”.

**Figure 32:**
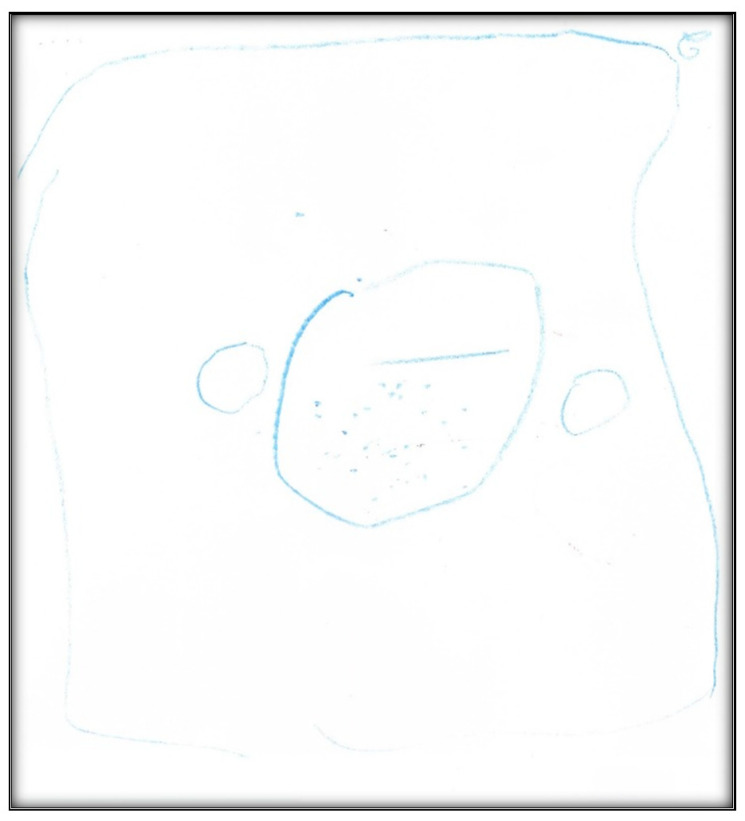
Child 7 Drawing 2 (pre-op).

**Child 7’s drawings.** The child drew two pictures depicting her situation (Figure 31 and Figure 32). Child 7 is in the pre-schematic stage of drawing when objects are formed with circles, squares, and lines, and generally float on the paper without correct proportion [16]. In this stage, telling stories is an important part of children’s drawings, so we asked Child 7 to give meanings to her drawn symbols. The child narrated that she drew three teeth and a tooth fairy that looks like a butterfly. She told us the teeth and butterfly all are sick because they have “holes”.

**Figure 33:**
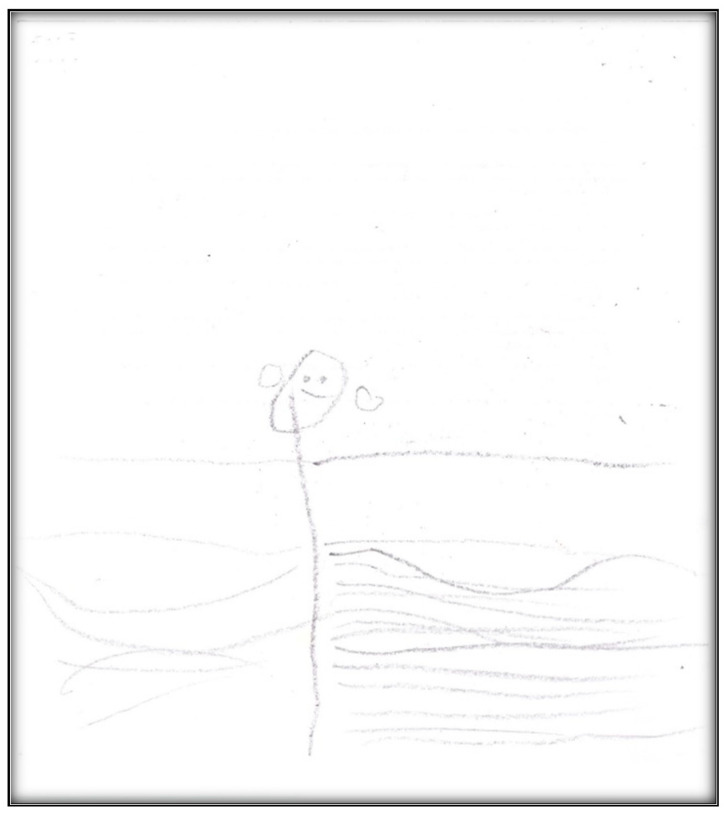
Child 5 Drawing 1 (post-op).

**Figure 34:**
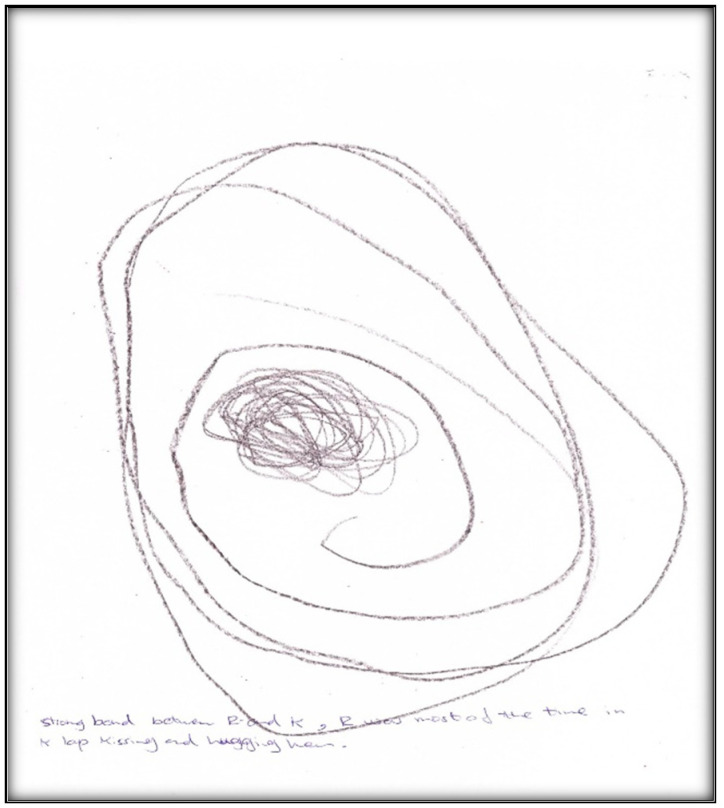
Child 5 Drawing 1 (post-op).

During the post-operative interview, Child 7 drew a two more pictures (Figure 33 and Figure 34). The first depicts a dream the child had right after surgery. The picture showed a “black dragon” with “a lot (of) arms” and a smiley face of her stuffed “Suzy Sheep”. The child was also talking about a bad smell and referred to it as “stinky garbage”. In the second drawing, the child scribbled some lines depicting a “tooth” that feels “OWEE” (in pain).

**Figure 35:**
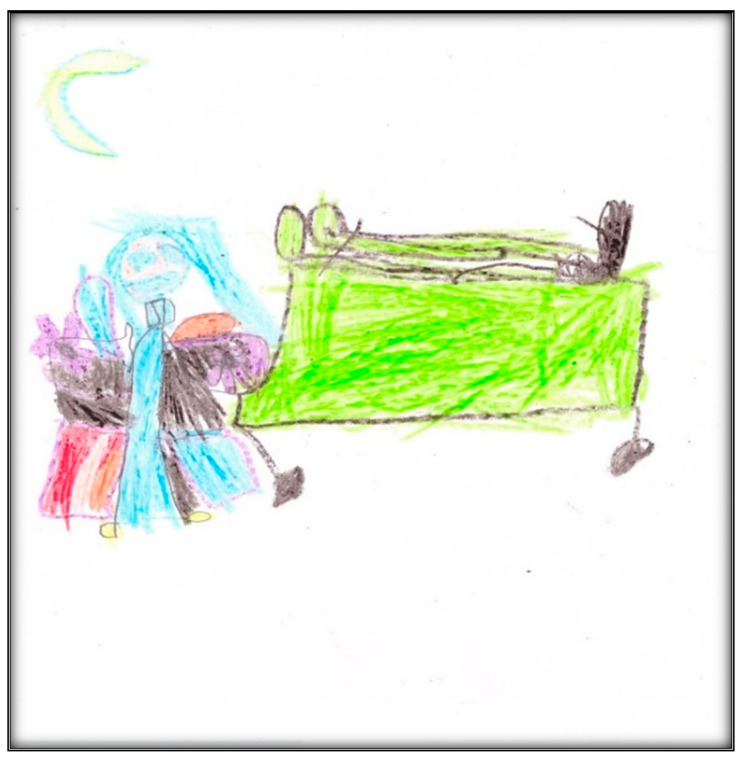
Child 8 Drawing 1 (pre-op).

**Figure 36:**
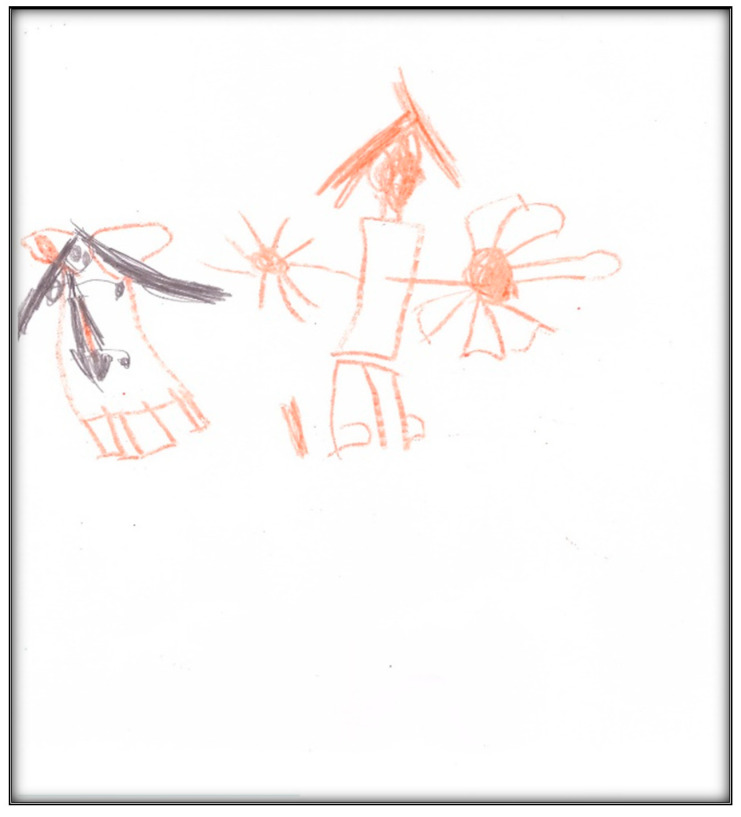
Child 8 Drawing 1 (pre-op).

**Child 8’s drawings.** The child drew two pictures (Figure 35 and Figure 36) depicting herself in a dental chair or lying in a hospital bed with either her mother or tooth fairy next to her. Child 8 made a connection between going to a dentist and the tooth fairy:

“Grip the tooth fairy. When somebody’s tooth falls out, they put it under the pillow. Then the tooth fairy comes when it is night, and that the tooth fairy makes them into a coin with her wand.”

Child 8 drew Figure 36 in black and brown, depicting a girl, who “has her teeth out”, in a dental chair and the mother’s hand next to her. In comparison, the colors in drawing the tooth fairy in Figure 35 were multiple; the yellow color was used to depict the moon. The *Child-Drawing: Hospital* rating scale considers black and brown to be associated with more stressful events; yellow, green, and blue are associated with less stressful events. Additionally, the fewer colors used, the more stressful the event. Figure 35 shows Child 8, asleep and covered. The drawing shows two bed legs facing the viewer, whereas the other two bed legs (away from the viewer) were missing. This feature might represent the developmental level of Child 8.

**Figure 37:**
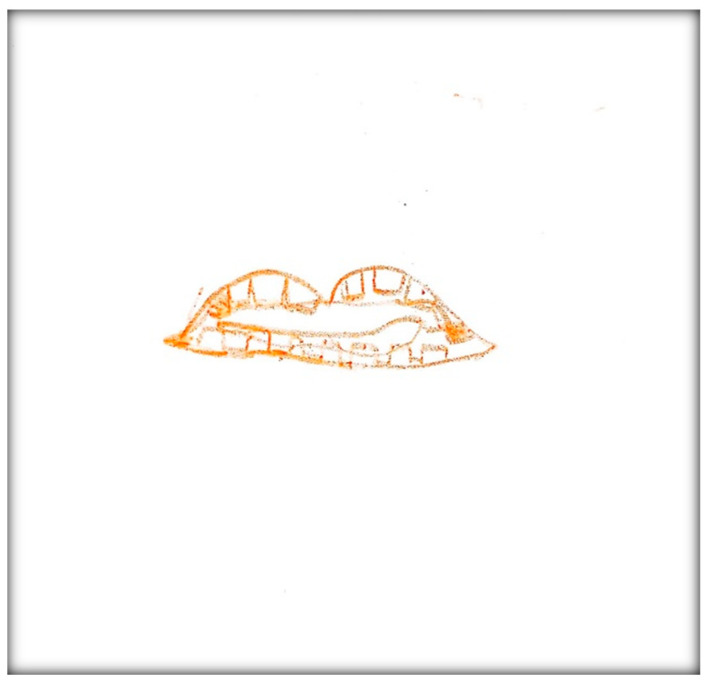
Child 9 Drawing 1 (pre-op).

**Figure 38:**
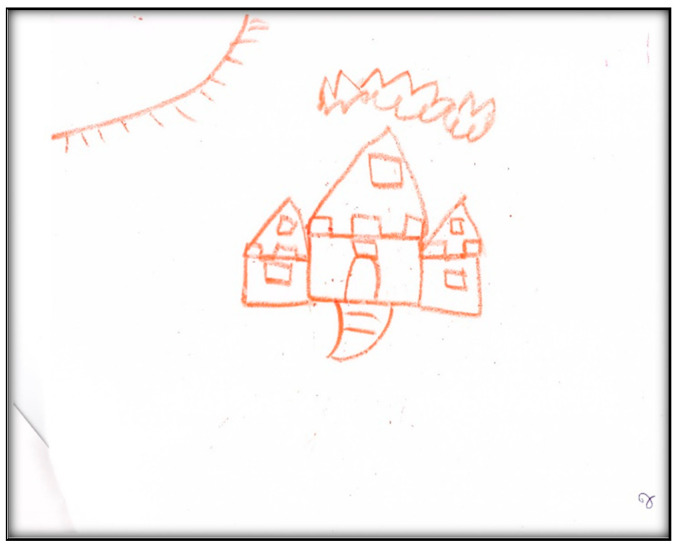
Child 9 Drawing 2 (pre-op).

**Child 9’s drawings.** During the pre-operative interview, the child drew two pictures. Figure 37 depicts his mouth, teeth, and tongue. He commented “this tooth has a cavity”. Figure 38 depicts the sun, clouds, and the townhouse where he lives. When asked why he drew the sun, he replied: “To give us some heat”. It is worth noting that both pictures were drawn in red, a color characteristic of intense anger, aggression, threat, fear, and loss of control, frequently found with increased anxiety. Another item of note is the child did not draw anything related to his dental experience at the dental offices, indicating a probable need for denial.

**Figure 39:**
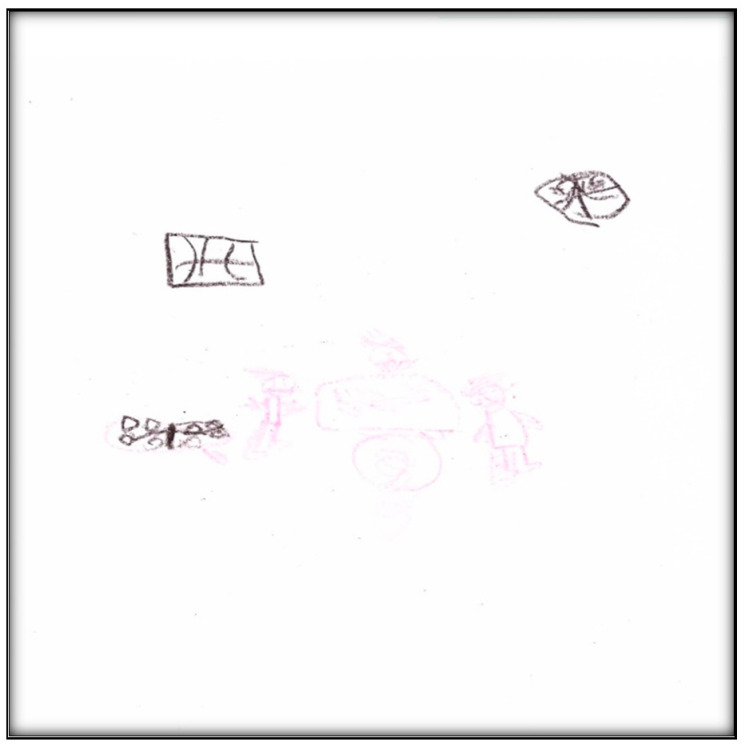
Child 9 Drawing 1 (post-op).

**Figure 40:**
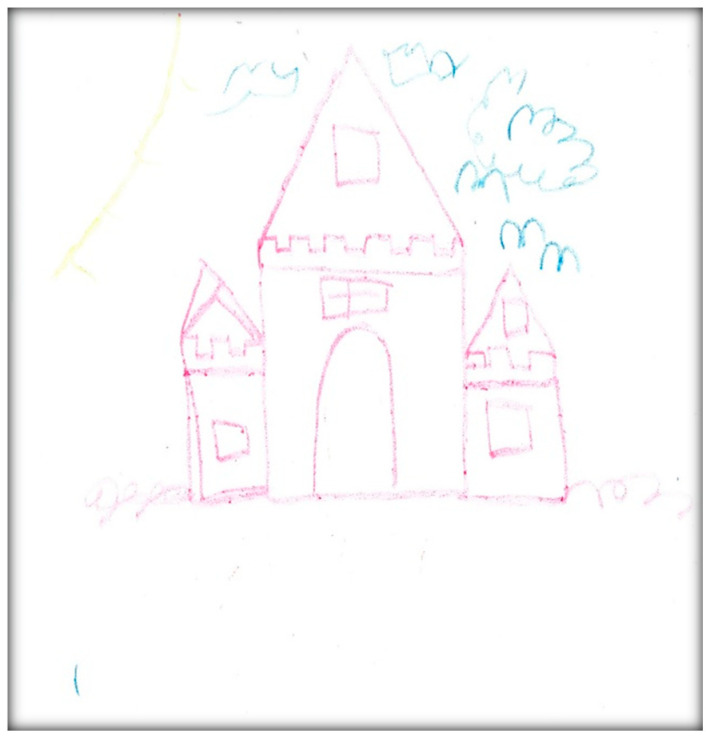
Child 9 Drawing 2 (post-op).

**Figure 41:**
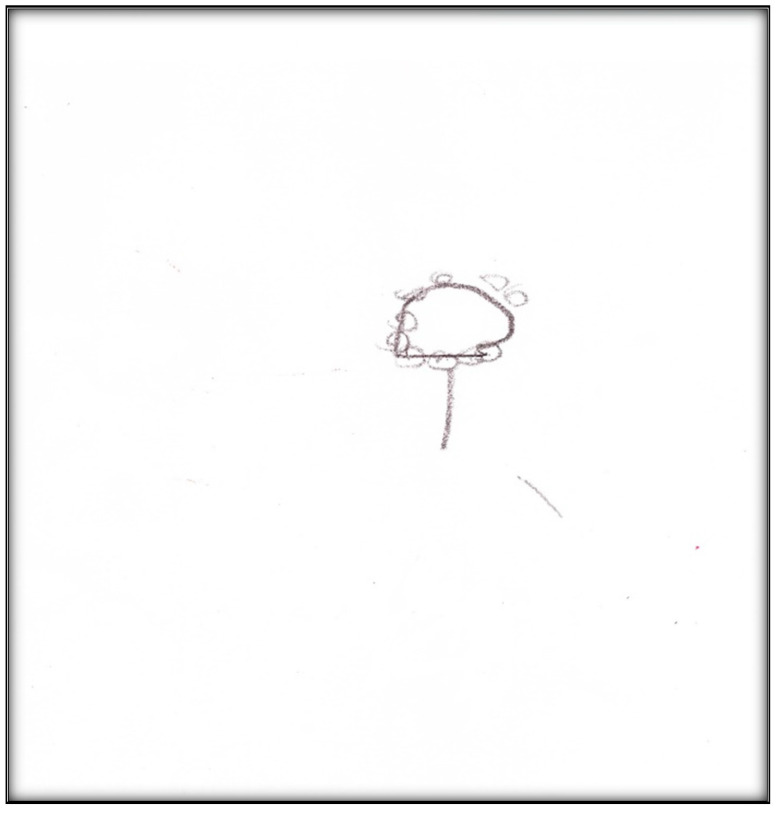
Child 9 Drawing 3 (post-op).

Child 9’s drawings (Figure 39, Figure 40 and Figure 41) 2 weeks after dental surgery mirror most of the concerns discussed with the family. In the first drawing, he drew a home (Figure 39). The second drawing (Figure 40) depicts an operating room where he was lying on a bed, along with the mask and two doctors, next to him, as well as, more importantly, his nine extracted teeth. The OR in this picture has two windows. In the last drawing (Figure 41) the child drew nine extracted primary teeth as leaves falling off a rose.

Child 9 drew the OR in red and black. Both colors are characteristic of intense anger, feeling threatened, fear, and loss of control frequently associated with increased anxiety. The drawing shows the child in a state of submission, and has medical personnel (all are distorted, ghost-like creatures) and equipment. The drawing also represents the height and size difference between the professional and patient, showing a power relationship. In the drawings, the child also expressed the emotional pain he suffered seeing his nine teeth removed from his body. The child portrayed not only his emotional pain, but also what he could see through the OR windows, which may suggest optimism beyond the dental treatment.

When asked to draw about dental treatment, Child 9 drew homes (in both interviews). The house in both drawings was huge with windows, images of a sun and clouds, all representing love and life. This might suggest a safe place, in contrast to the situation the family left to move to Canada.

**Figure 42:**
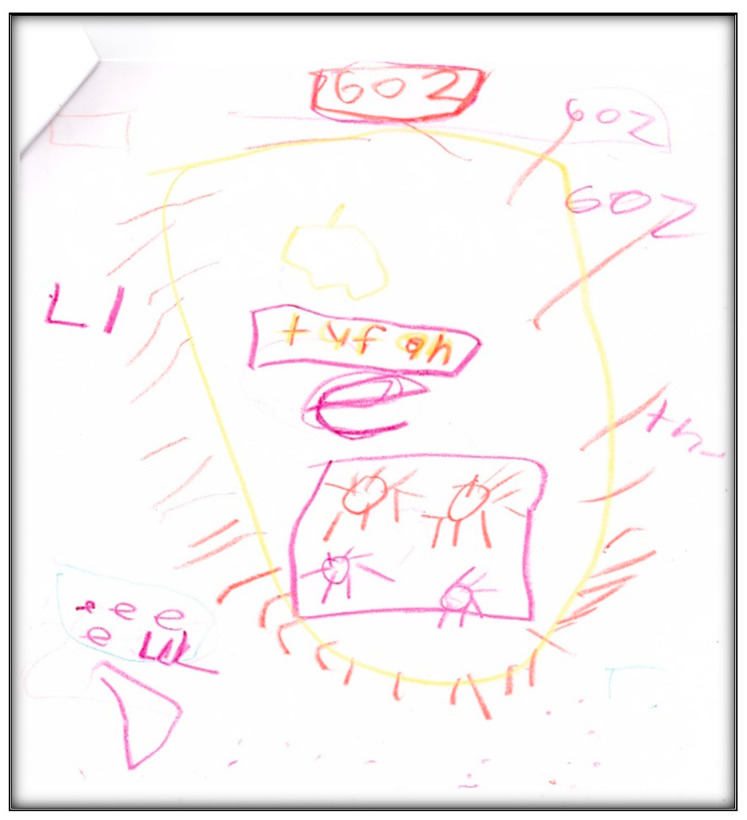
Child 10 Drawing 1 (post-op).

**Child 10’s drawings.** The child drew a picture depicting his teeth, four suns, and an apple core (Figure 42). He also jotted down the number of his room (602) three times. It is worth mentioning that Child 10 lisps now, related to the extraction of his upper anterior teeth. The mother clarified what he was saying several times during the interview. For example, he was referring to his “seewii teeth” meaning silly teeth. The removal of his teeth also affected his ability to eat apples, hence the representation of an apple in the drawing.

**Child 11’s drawing.** Although we left papers and crayons at his house for a few days, the child (8 years) did not want to draw anything related to his dental experience. The drawings collected were completed by his siblings and were not included in the analysis.

**Figure 43:**
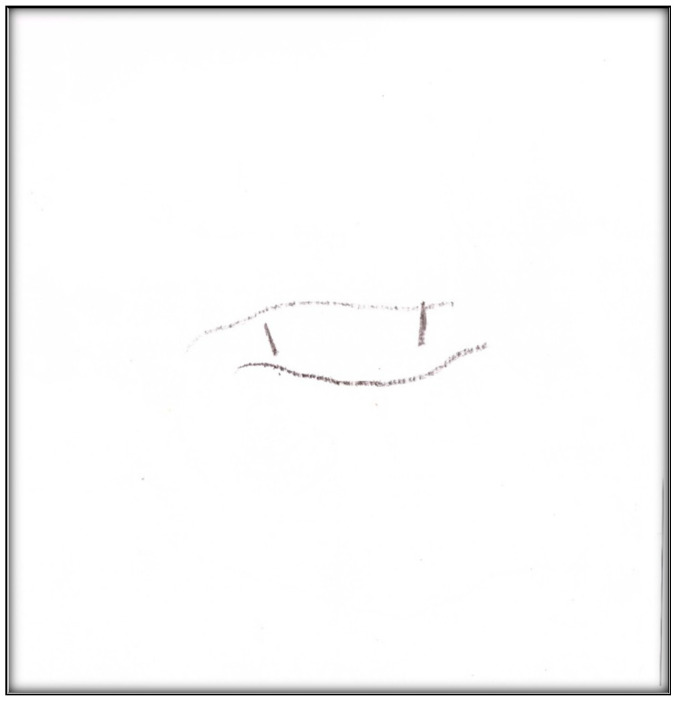
Child 12 Drawing 1 (post-op).

**Child 12’s drawing.** Although only 3 years old, the interview was informative as the child expressed herself independent of her mother. She even drew an oral cavity with two teeth inside (Figure 43). She was able to remember and narrate fine details from her GA, although it had been several weeks since surgery. The child described the appearance of the surgery center (“there was a picture with a girl playing and flowers”), its color (“green”), and the smell (“sweet”) of the balloon (i.e., surgical mask) used to induce her. The child repeated her concern about the removal of her teeth during the interview (“They removed my teeth”).

Table 3 presents the main physical and psychological impacts during the three periods (pre, peri-, and post-operative) comprising the care pathway, along with the words that the participants actually used to describe the various impacts. The words used to describe oral health and teeth are also presented. In the last row of the table, “what the child got out” of this experience is tabulated.

## 4. Discussion

Engaging children’s perspectives is challenging, and drawings can open a child-centric dialogue about matters important to them. The premise is, “Children’s ability to retrieve information that is encoded about their experiences may be more readily accessed by stimulating their perceptive senses than by semantic stimulus”, and that participatory methods (e.g., story games, drawings, scrapbooks, mapping, photographs, and videos) can be used as innovative and developmentally appropriate methods to visualize health, and to reveal how children understand illness and communicate their experiences [22].

In the current study, drawings by the children were used as a visual medium to complement their verbal responses. Drawings enhanced the spoken word and often gave a richer, more holistic understanding of participants’ worlds, as well as often acting as stimuli. Broadly, visual material such as drawings have a role in two aspects of research: they can be a form of data gathered from research participants and initiated either by the researcher or by the research participants; alternatively, they can be used as a stimulus that is provided by the researcher to act as a prompt or focus of discussion. However, these two aspects are not discrete and often overlap as seen here. In short, drawings were used to understand children’s lives and experiences in their voices and engaged children regarding their own perspectives on their dental concerns.

While it is true that exodontia (i.e., removing teeth) services under GA require less time and equipment, thus representing a less expensive option than provision of comprehensive care, children in this study (and some of their parents) reported extraction of teeth as negative outcomes, being distressed by this loss. This loss was represented in some children’s drawings in the current study by images of the tooth fairy. For example, Child 5 drew the tooth fairy as a beautiful little child with wings flying through the air. Some children pictured something else in place of a tooth fairy (e.g., a dog by Child 4; a bear by Child 5; a “Girl Picachu” by Child 4). Notably, among gift-bearing imaginary figures, only the tooth fairy (compared to Santa Claus and the Easter Bunny) can appear in a variety of ways and can take on any appearance, enabling children to use their imaginations when describing her (Wells, 1991). However, some common characteristics of the tooth fairy include long hair, a long dress, wings, and a wand, in addition to being feminine (all features depicted in Child 8’s representation of a fairy).

Toumba (2013) suggests that dentists should better use the tooth fairy to promote the oral health of children’s teeth by telling children that they will get more money from the tooth fairy for sound teeth than for decayed teeth [23]. Parents who are often struggling to get children to brush their teeth could also use this type of persuasion.

This study shows that drawing can facilitate discussions with children as young as three, and help them talk about their traumatic experiences; the drawings provide a link between their internal thoughts and perceived reality. This is important because children, particularly young children, who are more frequently subjected to DTGA, may not always be able to verbally communicate their exact feelings, experiences, fear, and anxiety. While Thomas and Jolley (1998) [24] indicated that children’s drawings on their own are too complexly determined and inherently ambiguous to be reliable sole indicators of the emotional experiences of the children who drew them, we found that drawing gave children the opportunity to express clues that were internally generated and which helped children organize their narratives and share their story. It is worth noting that, in this study, children showed a great interest and tendency to draw (except one child who did not want to make a drawing). Child 5 (age: six years and five months) drew a total of 14 drawings during the three time periods of the study: pre-operative (four drawings), peri-operative (two drawings), and post-operative (seven drawings). In addition, this child recorded two video diaries, both the night before her GA appointment. The drawings, the video diaries, and the narratives enabled the children to tell their stories, which benefited this research tremendously. The drawings can also serve as a reward for children, in addition to being a tool to establish rapport with them to facilitate communication and bond. Several children were proud of their drawings, signing them. Although analyzing the drawings was based on theories, the methods used here are valid and supported by empirical evidence generated from medical, dental, and educational settings [25,26]. That being said, further studies with larger sample sizes are recommended to shed more light on this novel research approach in different dental settings.

## 5. Conclusions

This study underscores the importance of using qualitative data to understand the lived experiences of children undergoing dental treatment, and how drawings can be used as a means of generating rich data on children’ experiences—in this particular case, with DTGA.

## Figures and Tables

**Table 1:** Child Participants.

Code	Age	Sex	Race/Status	Age of 1st Dental Visit	Pain	Payment	DTGA
Child 1	5.8	F	Indigenous	2 y	Yes	Government	2 extractions 3 SSCrowns/other crowns 2 amalgams Prophy and fluoride
Child 2	8.8	F	Indigenous	4.5	Yes	Government	1 pulp treatments 1 SSCrowns/other crowns 1 amalgam Prophy and fluoride
Child 3	6.3	F	Caucasian	3	Yes	3rd party	1extraction 2 pulp treatments 3 SSCrowns/other crowns Prophy and fluoride
Child 4	9.9	F	Indigenous	2	Yes	3rd party	2 SSCrowns/other crowns 1 amalgam 4 composites Prophy and fluoride
Child 5	6.5	F	Caucasian	4	No	3rd party	1 extraction 1 pulp treatment 3 SSCrowns/other crowns 4 amalgams Prophy and fluoride
Child 6	2.6	M	Indigenous	1	No	Government	3 pulp treatments 8 SSCrowns/other crowns 1 composite Prophy and fluoride
Child 7	4.3	F	Indigenous	4.2	Yes	3rd party	1 extraction 4 pulp treatments 5 SSCrowns/other crowns 1 amalgam 4 composites Prophy and fluoride
Child 8	5.2	F	Caucasian	1.5	No	3rd party	no treatment
Child 9	6.5	M	Refugee	6	Yes	Government	9 extractions (OR) 2 or 3 extractions (clinic) 4 amalgams 4 SSCrowns
Child 10	6.6	M	Newcomer	3	No	Government	8 extractions 8 SSCrowns 2 Band & Loop
Child 11	8.1	M	Refugee	5	Yes	Sponsor	N/A
Child 12	3.1	F	Refugee	2	Yes	Government	N/A

DTGA: dental treatment completed under general anesthesia, F: female, M: male.

**Table 2:** Sample Child Drawing: Hospital Score Sheet.

	Score	Description
Part A		
Person: Position	10	Dependent position (i.e., person lying in bed) with grounding
Action	10	Rigid figure, no arms
Length of person	7	Very small constricted person who appears overwhelmed by the paper
Width of person	7	Stick figure, no clothing
Facial expression	10	No face, no expression
Eyes	10	No eyes
Size of person to environment	8	Very small
Color: Predominance	8	Red (characteristic of intense feelings of threat, fear, and loss of control)
Color: Number used	9	Two colors only
Use of paper	9	Restricted 1/4–1/8 of the sheet
Placement	7	Left-sided placement
Strokes: Quality	8	All strokes light. Some may be firmer than others.
Hospital equipment	1	None included
Developmental level	7	Below normal (head, eyes, mouth, arms missing)
Total Part A	111	
**Part B**		
Add 10 points for omission of two or more parts	10	Omission of more than two parts
Add 10 points for distortion	10	The whole body is misshapen
Total Part B	20	
Part C		
Overall sense of the picture (gestalt rating)	5	Less pleasant, some distortion of size, less cheerful
Total score A + B + C	136	Above average level of anxiety (130–167)

**Table 3:** Descriptors Used by the Study’s Participants during the Pathway of Care.

Time	Impact	Descriptors Used
Pre-, peri-, and post-operative	Anxiety	“Worried”, “Scared”, “Nervous”, “Anxious”, “Horrifying”, “Felt guilty”, “Afraid”, “Unsettling”, “Suffering”, “Upset”, “Angry”, “A pretty tighten”, “Cry”, “Kind of scared”, “Anticipation”
	Fasting	“Hungry”, “Thirsty”, “Dry mouth”
	Anesthesia	“Put on sleep”, “Going to sleep”, “Fall asleep”, “Numb”, “Fuzz”, “Fishistalia [i.e., anesthesia]”, “Traumatized to needles”, “Don’t feel anything”, “Blow something on my head” (the mask), “Go to sleep in no time”, “Inhaled gas”, “Froze” (hand for IV)
	Smell and surgical mask	“Stink”, “Plastic”, “Canadian tire”, “Rubber”, “Blow up a balloon”, “Take it [mask] off”, “Gross”
	Nausea	“Sick to my stomach”, “Nausea”, “Sick”, “Nauseous”, “Threw up”, “Pukey”, “Vomit”
	Cannula	“IV pressure”, “Needles”, “Bruise” (skin), “Poke”
	Edema	“Stuffed” (face), “Look like a vampire”, “Swelling”
	General descriptors	“Bored”, “Drowsy”, “Annoyed”, “Druggie” (appearance), “Make a beep” (calm), “Grumpy”, “Squirming” (child), “Wake-up room” (recovery), “Pale”, “Yucky” (medicine), “Dry socket”, “Cranky”, “Tired”, “Twitchy”, “Dizzy”, “Sore” (chest), “Distract” (after GA), “Bleeding”, “Weird”, “Not herself”, “Dangerous [GA]”, “Fever”, “Mad”, “Super sore”
Oral/Dental Health		
	Dental trauma	“Knocked loose”
		“Wiggly”
		“Nerve dead”, “Abscessed tooth”
		“Turn dark”
		“Rotten teeth”
		“Sharp-edged” (tooth)
		“Bumped” (a tooth)
		“Pull out”, “Taken out”, “Extracted”
	Tooth preparation for a cap	“Shaving around”, “Drilling”
		“Tickling” (teeth)
		“Cap”
		“Crown”
	Dental decay	“Cavity”
		“Gray line”
		“Hole” (in a tooth)
		“Tight teeth” (contact area)
		“Cavities between teeth” (proximal caries)
		“Kissing cavities” (proximal caries)
		“Bad teeth” (easy to get cavities), “Toothaches”
	Terms to describe teeth	“Bottom tooth”, “Tooth in the back” (Molars)
		“White”, “Whiter”
		“Temporary teeth”, “Baby teeth”, “Big teeth”
		“Adult teeth”
		“Fuzzy”, “Sensitive”, “Bugging”
	Preventive	“Cover” (pits and fissures sealants)
		“Preventative”
	General dental terms	“Chew”, “Bite”, “Chunky” (food), “Gums”, “Stick finger in” (mouth due to pain)
What child “got out” of it	Positive impacts	“I got a teddy bear!”, “Ice cream after the surgery”, “Stuffed animal”, “Suzy Sheep”, “Tooth Fairy”, “Two dollars!”, “Get things from Walmart!” “Go to Burger King!”, “Go to Dollarama!”, “Days off school!”, “No more pain”, “No more toothaches”, “Stayed on the couch and watched TV”, “Relieved”, “Happy it was done”, “Glad it was over”
